# Aptamer-mediated liver-targeted curcumin delivery system based on tetrahedral framework nucleic acids for NAFLD

**DOI:** 10.1080/10717544.2025.2576222

**Published:** 2025-11-09

**Authors:** Shaoyun Chen, Yuchen Liu, Siying Ma, Lin Chen, Liping Zhou, Jiawen Wang, Yingying Huang, Zhiling Yu, Xiaobing Dou

**Affiliations:** a School of Life Sciences, Zhejiang Chinese Medical University, Hangzhou, Zhejiang, People's Republic of China; b School of Pharmaceutical Sciences, Zhejiang Chinese Medical University, Hangzhou, Zhejiang, People's Republic of China; c School of Medicine, Zhejiang University, Hangzhou, Zhejiang, People's Republic of China; d School of Chinese Medicine, Hong Kong Baptist University, Hong Kong, People's Republic of China; e Zhejiang-Hong Kong Joint Laboratory of Liver and Spleen Simultaneous Treatment in Traditional Chinese Medicine, Traditional Chinese Medicine, Hangzhou, Zhejiang, People's Republic of China

**Keywords:** Curcumin, tetrahedral framework nucleic acids, aptamer, NAFLD

## Abstract

Curcumin is renowned for anti-inflammatory, antioxidant and hepatoprotective effects, and has been implicated in the amelioration of obesity and diabetes. Notwithstanding its considerable therapeutic potential, the clinical utility of curcumin is hampered by its suboptimal bioavailability, due to poor aqueous solubility and chemical instability. Consequently, the development of strategies to enhance the aqueous solubility, stability, and ultimately, the bioavailability of curcumin has been a focal point of intense research. This study harnessed tetrahedral framework nucleic acids (tFNAs), a relatively simple DNA nanostructure, to encapsulate curcumin. Meanwhile, novel aptamers for liver-specific targeting were acquired by SELEX (Systematic Evolution of Ligands by Exponential Enrichment) method. By capitalizing on the unique properties of aptamers and tFNAs, an aptamer-mediated liver-targeted curcumin delivery system was constructed, with the goal of providing a more efficacious therapeutic approach for non-alcoholic fatty liver disease (NAFLD). This innovative delivery platform has not only markedly improved the solubility and stability of curcumin but has also significantly bolstered its therapeutic efficacy in the context of NAFLD. This research not only offers a novel approach for the delivery of curcumin but also presents a new therapeutic modality for NAFLD. Moreover, the implications of this research extend beyond curcumin, offering a blueprint for the liver-targeted delivery of other drug molecules.

## 1. Introduction

Curcumin, a natural polyphenolic compound, functions as the principal active constituent in turmeric (*Curcuma longa*) (Jabczyk et al. 2021). The presence of diketone, phenolic groups, and two aryl rings endows curcumin with a diverse spectrum of pharmacological activities (Jabczyk et al. 2021; Zeng et al. 2023). It demonstrates a wide variety of beneficial properties, including antioxidant (Keramat et al. 2024), anti-inflammatory (Feng et al. 2025), hepatoprotective (Yashmi et al. 2024), neuroprotective (Baghcheghi et al. 2025), anticancer (Hao et al. 2024), antibacterial (Sharma et al. 2024), and antiviral activities (Sharma et al. 2024). Additionally, it can reduce the occurrences of diabetes, obesity, as well as cardiovascular and cerebrovascular diseases (Jabczyk et al. 2021; Mahmoudi et al. 2022; Zeng et al. 2023). Furthermore, curcumin has the capacity to activate multiple signaling pathways, such as adenosine 5'-monophosphate-activated protein kinase (AMPK) and nuclear factor-kappa B (NF-κB), while suppressing oxidative stress and cell apoptosis (Li et al. 2024); thereby, it alleviates the lipid metabolism disorders associated with non-alcoholic fatty liver disease (NAFLD) (Safari et al. 2023).

Despite curcumin’s favorable medicinal properties, its clinical application encounters significant hurdles primarily due to its poor bioavailability (Pawar et al. 2024). Thus, curcumin is poorly absorbed, rapidly metabolized, and quickly eliminated from the body (Pawar et al. 2024). This limits the concentration of curcumin in the target tissues, thereby reducing its therapeutic effectiveness (Pawar et al. 2024). Encapsulation or formulation of curcumin into nano-scale structures is a commonly adopted approach to enhance its bioavailability and pharmacotherapeutic activity (Gayathri et al. 2023). A variety of nano-formulations, including nano-emulsion, polymeric nanoparticles, solid-lipid nanoparticles, metal nanoparticles, and liposomes, have been developed for curcumin drug delivery (Gayathri et al. 2023). Moreover, the utilization of curcumin nanoparticles (Yadav et al. 2019), nanogels (Kaewruethai et al. 2023), liposomal encapsulation (Hasan et al. 2019; Hegde et al. 2023), curcumin-phospholipid complexes (Hassanizadeh et al. 2023; Song et al. 2022), as well as adjuvants such as chitosan, *β*-cyclodextrin, PEG, and surfactin (Hasan et al. 2019; Shan et al. 2022; Song et al. 2022) has been verified to improve curcumin’s bioavailability. For more efficient curcumin delivery, several targeted delivery strategies have also been adopted. For instance, the carboxymethylcellulose ester of curcumin was developed for a sustained release delivery system in the liver (Mor et al. 2023). Sodium alginate coatings and dendritic copolymers, featuring pH-responsive release characteristics and dual-release rates, have been successfully utilized for targeted transport of curcumin to cancer sites (Chai et al. 2025).

Recently, DNA nanostructures, such as tetrahedral, cruciform, nanoflower, nanostar, nanocentipede, and nanococklebur, have been regarded as a powerful precision drug delivery system (Safarkhani et al. 2024). DNA nanostructures can be synthesized by different methods, including the DNA Origami technique and DNA Brick Assemblies (Jiang et al. 2024; Safarkhani et al. 2024). They possess rationally pre-designed geometries, precise addressability, and versatile programmability (Jiang et al. 2024). Moreover, DNA, being a biological endogenous substance, exhibits excellent biocompatibility, which further augments its potential as a drug delivery carrier (Han et al. 2023).

Tetrahedral framework nucleic acids (tFNAs), as relatively simple DNA nanostructures, were successfully fabricated through programmed self-assembly and initially reported in 2005 (Goodman et al. 2005). In addition to their outstanding cargo-loading capacities, their remarkable plasma-membrane permeability, high stability, and the absence of significant adverse effects make them particularly attractive in the rapidly growing biomedical research field (Zhang et al. 2020). Lin and co-workers utilized tFNAs to encapsulate wogonin in vitro and formulated a tetrahedral framework nucleic acid–wogonin complex, which effectively mitigated the inflammatory response and prevented cartilage damage during the treatment of osteoarthritis (Shi et al. 2020). In the following year, another study led by Li et al. (2021) once again employed tFNAs to load resveratrol. This approach not only effectively enhanced the water solubility of resveratrol but also improved its stability, thereby controlling the tissue inflammation process during the onset and progression of insulin resistance (Li et al. 2021). These research efforts inspired us to consider applying tFNAs for curcumin delivery.

Targeted drug delivery to specific sites has always been regarded as a valuable strategy to enhance therapeutic efficacy and minimize side effects (Komiyama et al. 2023). Nucleic acids have emerged as programmable materials for the development of targeted delivery systems, thanks to their inherent self-assembled nature (Xuan et al. 2024). In response to external environmental stimuli, DNA response elements can act as switches to initiate conformational alterations in DNA structures (Xuan et al. 2024). Notably, aptamers, which possess high affinity and specificity for particular targets, have rapidly emerged as a new class of targeted ligands utilized in drug delivery (He et al. 2023). Recently, the incidence of liver-related diseases has been increasing annually, yet the corresponding treatment measures remain relatively scarce. This has drawn significant attention to the application of aptamers in liver diseases (Xu et al. 2024). However, reports on aptamer-mediated drug delivery systems for liver targeting are rather scarce.

Consequently, this study aimed to employ the SELEX (Systematic Evolution of Ligands by Exponential Enrichment) method (Chinchilla-Cárdenas et al. 2024) to screen novel aptamers and enrich the aptamer library for liver-specific targeting. Meanwhile, tFNAs were utilized to encapsulate curcumin. By capitalizing on the unique properties of aptamers and tFNAs, a meticulously engineered liver-targeted curcumin delivery system was constructed, with the goal of providing a more efficacious therapeutic approach for non-alcoholic fatty liver disease (NAFLD).

## 2. Materials and methods

### 2.1. Materials

All DNA sequences were synthesized by Shanghai Saiheng Biotechnology Co., Ltd. All chemicals were acquired from standard commercial sources. Curcumin was purchased from Hangzhou Bangyi Chemical Co., Ltd. Male C57BL/6 mice were purchased from Shanghai Slake Experimental Animal Co., Ltd. (License number: SCXK (Shanghai) 2017-0005). All experimental animals in this study were handled in accordance with the animal experimental guidelines approved by the Animal Ethics Committee of Zhejiang Chinese Medical University (Animal Ethics Approval Number: IACUC-20210830-09). AML12 (mouse hepatocyte) (CL-0602) and RAW 264.7 (mouse mononuclear macrophage) (CL-0190) cells were purchased from Pricella Biotechnology Co., Ltd. GES-1 (human gastric mucosal epithelial cell) (GX-0080) cell was purchased from BOSTER Biological Technology Co., Ltd.

### 2.2. Drug preparation

In multiple 2-mL centrifuge tubes, ddH₂O was added in specific volumes: 750 μL for Groups VI and VIII, and 600 μL for Groups V and VII. Subsequently, 150 μL of magnesium chloride solution (20 mmol/L), 150 μL of TF1 (10 μmol/L), 150 μL of TF2 (10 μmol/L), and 150 μL of TF3 (10 μmol/L) were sequentially incorporated. Then 150 μL of TF4A (10 μmol/L) was added into Groups VI and VIII, while 150 μL of TF4B (10 μmol/L) was added into Groups V and VII. Each mixture was then thoroughly vortex-mixed to ensure homogeneity. In Group V and VI, an excessive quantity of curcumin was introduced separately. The mixtures were then subjected to heating in a metal bath at 90 °C for a duration of 10 minutes. During this period, they were frequently vortex-agitated to maintain uniform distribution. Immediately following heating, the tubes were rapidly cooled in an ice bath for 2 minutes to quench the reaction, and then centrifuged at 5000 r/min for 1 minute. The resulting supernatants were carefully pooled into a 15-mL centrifuge tube. For the drugs in Group V and VII, M15BT, which had been pre-treated by denaturation at 95 °C for 5 minutes followed by natural cooling for annealing, was added in an amount equivalent to that of TF4B. The mixtures were vigorously shaken to ensure complete mixing and then allowed to incubate statically at 37 °C for 1 hour.

A 50-μL aliquot of the drug solution containing curcumin was transferred, and 450 μL of methanol was added. The resulting mixture was thoroughly mixed and then analyzed by HPLC (High Performance Liquid Chromatography) to determine the curcumin concentration. To adjust the curcumin concentration to 50 μg/mL, an appropriate volume of the drug solution from Group VII was added to the solution in Group V. Similarly, the drug solution from Group VIII was used to adjust the curcumin concentration in Group VI to the same level.

### 2.3. HPLC detection of curcumin

Liquid chromatographic column: Welchrom® C18, UF-AA, 250 mm × 4.6 mm × 5 μm. The mobile-phase composition was set as a 6:4 ratio of acetonitrile to 0.1% formic acid in water. The flow rate was maintained at 0.8 mL/min, the detection wavelength was set at 425 nm, and the temperature was 25 °C. By taking the total area of the three curcumin-related peaks, multiplied by 10^−5^, as the y-values, and the curcumin concentration (μg/mL) as the x-value, the standard-curve equation was determined to be y = 0.7187x, with a high-correlation coefficient of R^2^ = 0.9991 within the concentration range of 0 < x ≤ 120.

### 2.4. Grouping and administration of experimental animals

48 male C57BL/6 mice (20 ± 0.5 g) were housed within an SPF-grade barrier system. The housing environment was maintained with a 12-hour light and 12-hour dark cycle daily, an ambient temperature of 25 ± 2 °C, and a relative humidity of 55 ± 5%. After a one-week adaptive-feeding period, the mice were randomly assigned into 8 groups based on their body weights, with 6 mice in each group. The detailed animal-grouping and administration protocols are presented in Table 1. Starting from the second week, the administration process was carried out every two days. The mice in Groups III, IV, V, VI, VII, and VIII received tail-vein injections of the corresponding drugs at a dosage of 10 μL of drug per 1 g of body weight, calculated according to the individual mouse body weights. Meanwhile, the mice in Groups I and II were administered normal saline via the tail vein, following the same dosage regimen of 10 μL of normal saline per 1 g of body weight. The administration continued uninterrupted until the conclusion of the 13-week experiment. At the end of the study period, anesthesia was induced via intraperitoneal injection of 2% pentobarbital solution at a dosage of 0.2 mL per 100 g of body weight (to ensure the mice were fully relaxed but remained alive), and the mice were then prepared for tissue collection. Various animal tissue samples, including blood, liver, and fat, were collected for further analysis. Specifically, an incision was made in the abdominal region using tissue shears to expose the abdominal aorta. A disposable syringe was held in one hand, and the needle was inserted at an angle of less than 30° to the abdominal aorta. The needle was stabilized using the opposite hand, while the syringe was meticulously withdrawn to obtain the required volume of blood. Following the completion of blood collection, gentle pressure was applied to the needle insertion site with a cotton ball, and the needle was promptly removed. A volume of 0.8 mL of blood was collected from each mouse within a 10-minute timeframe. Terminal blood collection was succeeded by the confirmation of animal death through the administration of an anesthetic overdose. To minimize stress, all procedures were conducted under anesthesia.

**Table 1.** Grouping and administration of animal experiments.

Experiment	Group	Feed	Drug	Illustration
I	Normal Control	10%Low Fat Diet	Normal Saline	/
II	High–fat Model	60%High Fat Diet	Normal Saline	/
III	Ordinary Curcumin	60%High Fat Diet	50 μg/mL Curcumin, 1% DMSO	/
IV	Solvent	60%High Fat Diet	1% DMSO	/
V	Curcumin Encapsulated by Targeted tFNAs	60%High Fat Diet	50 μg/mL Curcumin, Targeted tFNAs	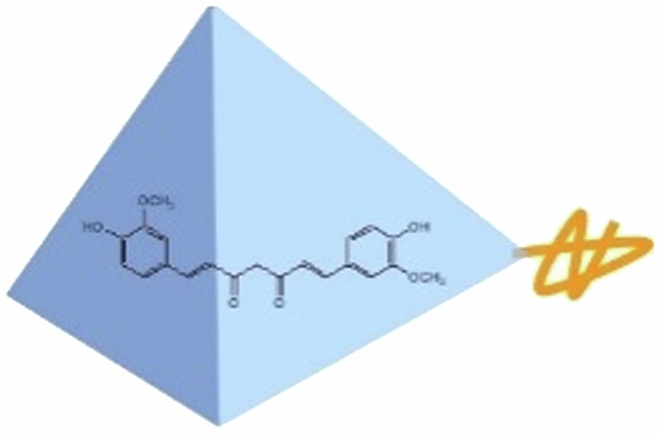
VI	Curcumin Encapsulated by Untargeted tFNAs	60%High Fat Diet	50 μg/mL Curcumin, Untargeted tFNAs	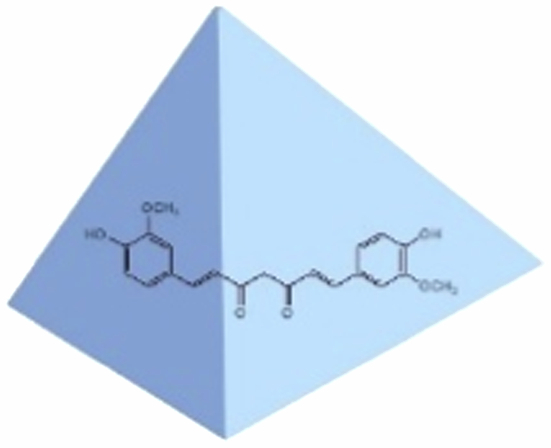
VII	No–load Targeted tFNAs	60%High Fat Diet	Targeted tFNAs	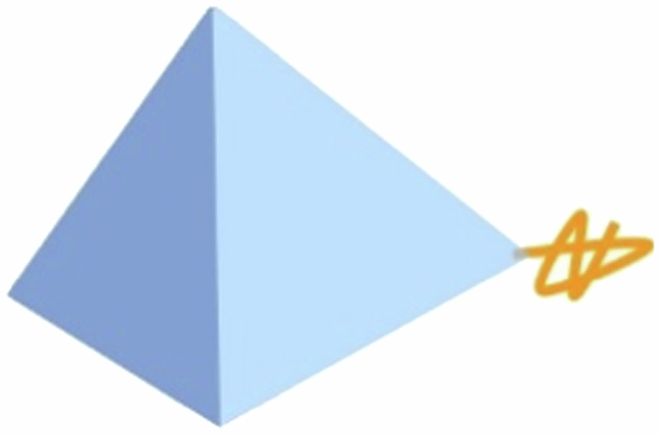
VIII	No–load Untargeted tFNAs	60%High Fat Diet	Untargeted tFNAs	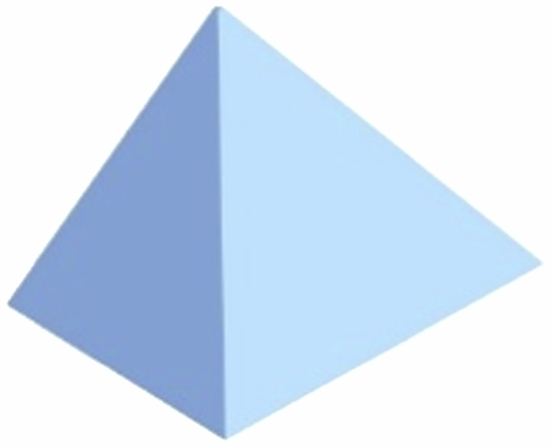

### 2.5. Statistical analysis

Data were presented from at least three independent experiments. Statistical significance was assessed using unpaired Student's t**-**test (for independent samples). All analyzes were performed using GraphPad Prism 9.0 (GraphPad Software, USA) or SPSS 26.0 (IBM, USA).

## 3. Results

Initially, a detection approach for curcumin was developed by HPLC. The HPLC chromatogram of curcumin is presented in Figure S1 (see Supplementary Information). Evidently, the methanol-dissolved curcumin sample exhibits three chromatographic peaks, with the main peak emerging at the latest elution time. This phenomenon can be attributed to the presence of three curcumin isomers (i.e. the three isomeric forms of curcumin). Simultaneously, it was observed that upon dissolving curcumin in water, the relative magnitudes of the three chromatographic peaks gradually undergo alterations. Specifically, the main peak diminishes while the other two peaks increase, as depicted in Figure S2(B) (see Supplementary Information). To mitigate the impact of isomer**-**related factors on quantitative analysis, the total area of the three curcumin peaks multiplied by 10^−5^ was designated as y, and the curcumin concentration (μg/mL) was set as x. Subsequently, a standard curve was constructed, yielding the equation y = 0.7187x (R^2^ = 0.9991, concentration range: 0 < x ≤ 120 μg/mL). Based on this analytical method, the water solubility of curcumin was further explored. It was determined that even after dissolving by heating at 90 °C for 10 minutes, its solubility in water remained below 5 μg/mL.

### 3.1. Principle design of liver–targeted delivery of curcumin

**Figure 1.**
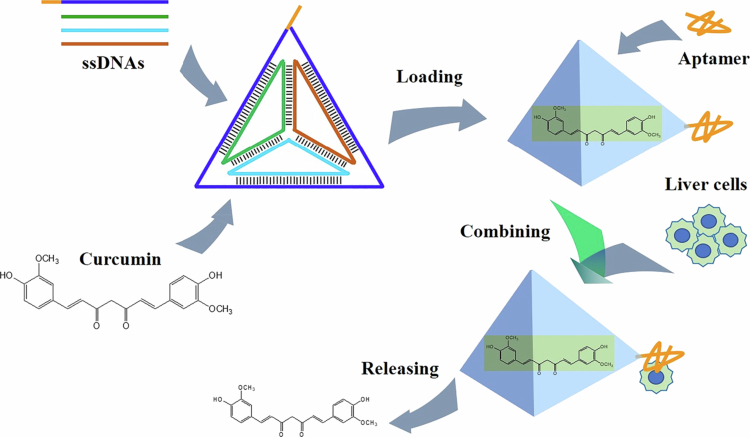
Schematic design of liver–targeted delivery of curcumin.

To augment the water solubility of curcumin and accomplish liver-targeted delivery, thereby enhancing its bioavailability, in this study, tetrahedral framework nucleic acids (tFNAs) were utilized to encapsulate curcumin, and a liver-specific aptamer was incorporated onto the tFNAs. The detailed process design is illustrated in Figure 1. tFNAs are assembled from four distinct single-stranded DNAs (ssDNAs). These tFNAs are capable of encapsulating curcumin, facilitating its dissolution and stability in an aqueous environment. Concurrently, one of the ssDNAs, namely TF4B, was deliberately designed to harbor the complementary sequence of the aptamer, with a melting temperature (T_m_) exceeding 50 °C. This enables TF4B to bind to the aptamer via base complementary pairing at room temperature, thereby completing the assembly of liver-targeted tFNAs. Owing to the excellent water solubility and biocompatibility of tFNAs, they can transport curcumin within the bloodstream. Subsequently, capitalizing on the liver-targeting property of the aptamer, targeted accumulation of the curcumin-loaded tFNAs in the liver can be achieved. Ultimately, under the action of endogenous biological nucleases, the tFNAs gradually degrade, releasing curcumin to exert its therapeutic effects.

### 3.2. Aptamer screening and specificity verification

**Figure 2.**
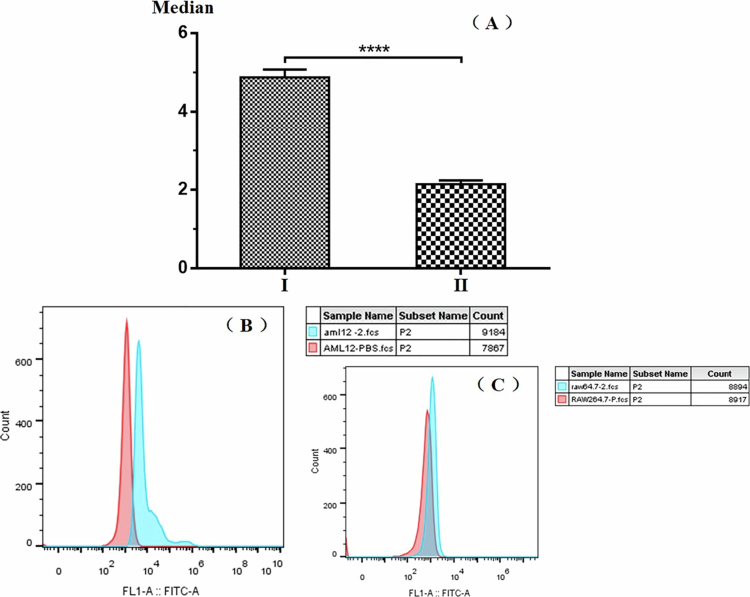
Specificity verification of aptamers. (A) Median. Ⅰ, AML12; Ⅱ, RAW264.7. (B) AML12. (C) RAW264.7.

Aptamers play a pivotal role in determining the targeting specificity of tFNAs. In this research, mouse hepatocytes AML12 were designated as the positive target, while mouse mononuclear macrophages RAW264.7 served as the negative target. Through the SELEX method, after 15 rounds of rigorous screening, the aptamer M15B was successfully acquired. The secondary structure of M15B was predicted using “The UNAFold Web Server”, and the resultant structure is depicted in Figure S3(A) (see Supplementary Information). Evidently, this aptamer is capable of forming a relatively stable hairpin-shaped secondary structure. A FAM fluorescent label was appended to the 3'-end of the aptamer M15B to synthesize the probe M15BF. Subsequently, M15BF was separately incubated with AML12 cells and RAW264.7 cells. Post-incubation and thorough washing, the fluorescence signal intensity was measured via flow cytometry. The outcomes are presented in Figure 2. When the M15BF probe binds to AML12 cells, a conspicuous disparity in fluorescence intensity between the sample and the background is observable. Conversely, when the M15BF probe binds to RAW264.7 cells, the difference in fluorescence intensity between the sample and the background is scarcely discernible. In terms of the median fluorescence intensity, a highly significant divergence (*p* < 0.0001) exists between AML12 cells (4.877 ± 0.2026) and RAW264.7 cells (2.143 ± 0.1002). This demonstrates that the screened aptamer M15B exhibits specific recognition for AML12 cells.

### 3.3. Construction of tFNAs

Four single-stranded DNAs (ssDNAs) were meticulously designed as a set. Each pair of ssDNAs in this set contains a 20-nucleotide (nt) complementary sequence with a melting temperature (T_m_) exceeding 50 °C. The assembly process began with high-temperature denaturation at 90 °C, followed by gradual annealing. During annealing, driven by base-pairing interactions between the complementary sequences, the four ssDNAs spontaneously self-assembled into a tetrahedral spatial architecture, thereby forming tFNAs. Specifically, TF1, TF2, TF3, and TF4B were mixed to construct Targeted tFNAs, while TF1, TF2, TF3, and TF4A were mixed to construct Untargeted tFNAs. The key difference between TF4B and TF4A is that TF4B is 25 nt longer, with 5 adenines (A) and a 20-nt tail at its 3'-end. This 20-nt tail serves as a crucial site for aptamer conjugation.

**Figure 3.**
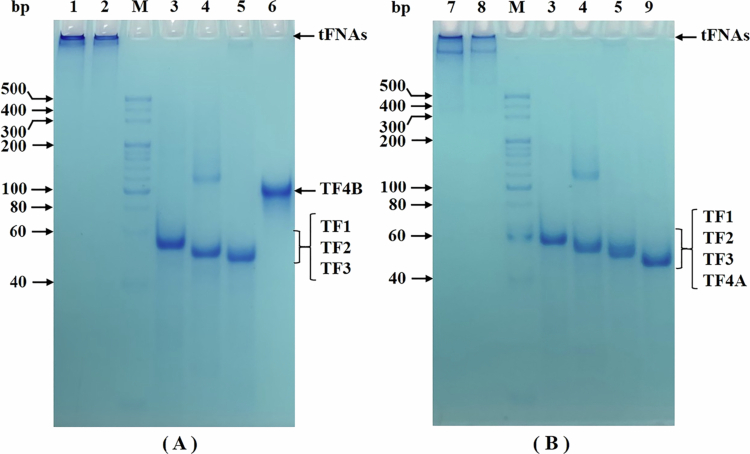
PAGE electrophoretogram of the tFNAs and ssDNAs. (A) Targeted tFNAs. (B) Untargeted tFNAs. M: Marker. Lane 1 and 2: Targeted tFNAs; Lane 3: TF1; Lane 4: TF2; Lane 5: TF3; Lane 6: TF4B; Lane 7 and 8: Untargeted tFNAs; Lane 9: TF4A.

The polyacrylamide gel electrophoresis (PAGE) profiles of tFNAs and their corresponding ssDNAs are presented in Figure 3. In Figure 3(A), due to the complex spatial conformation of Targeted tFNAs, they exhibit great difficulty in penetrating the gel matrix. Only a minuscule fraction of the structures entered the gel, as shown in lanes 1 and 2. A similar phenomenon was observed in the electrophoresis pattern of Untargeted tFNAs, as shown in Figure 3(B). Notably, slightly more Untargeted tFNAs entered the gel compared to Targeted tFNAs. This difference might be attributed to the 20-nt aptamer-binding tail in Targeted tFNAs: the tail not only increases the overall molecular weight of the tFNAs but also potentially enhances their structural stability-both factors could reduce gel penetration efficiency.

**Figure 4.**
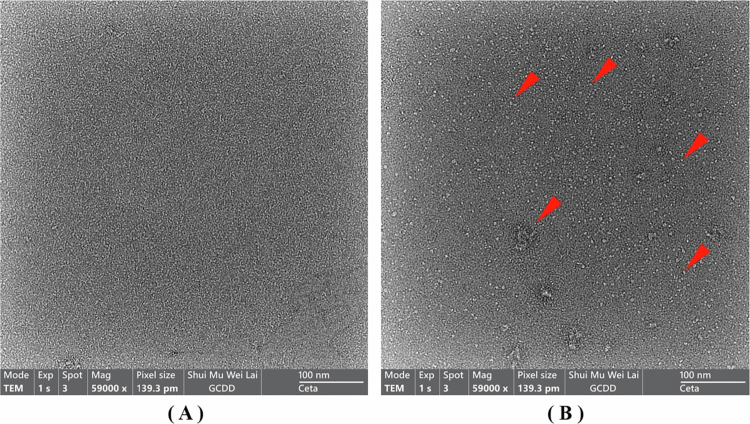
TEM photographs. (A) ssDNAs (TF1). (B) Targeted tFNAs.

To further verify the successful construction of tFNAs, transmission electron microscopy (TEM) was used to visualize the structure of Targeted tFNAs. The concentration of tFNAs was adjusted to 1 μmol/L. Since Targeted tFNAs are composed of 4 ssDNAs, the concentration of a single ssDNA sample was adjusted to 4 μmol/L to ensure equivalent total DNA content. As shown in Figure 4, a comparison between Figure 4(A) and (B) reveals numerous white dots in Figure 4(B) that are absent in Figure 4(A). These white dots are probably the tFNAs structures. A similar observation was made in the TEM images of Untargeted tFNAs, as shown in Figure S4 (see Supplementary Information). Furthermore, these characteristic white dots (prominent in tFNAs samples) were absent in the TEM images of single-ssDNA samples (TF2, TF3, TF4A, and TF4B; data not shown).

Subsequently, curcumin was encapsulated into tFNAs. A comparison between Figure S2(A) and S2(C) (see Supplementary Information) clearly shows that the color of the curcumin-containing solution deepened significantly after encapsulation with tFNAs. After storage at 4 °C for 24 h, HPLC analysis revealed that the curcumin concentration in the tFNAs-encapsulated solution exceeded 50 μg/mL-more than 10-fold higher than that in the ordinary curcumin aqueous solution. Further analysis of Figure S2(B) and S2(D) (see Supplementary Information) showed that the main HPLC peak of tFNAs-encapsulated curcumin maintained a stable size and shape, whereas the main peak of the ordinary curcumin aqueous solution decreased significantly. These results indicate that tFNAs-mediated encapsulation not only improves curcumin solubility but also enhances its structural stability.

Furthermore, the aptamer M15BT was designed for conjugation to Targeted tFNAs. An 18-nt tail was appended to the 3'-end of the previously screened aptamer M15B to generate M15BT. This 18-nt tail is complementary to the tail of Targeted tFNAs, with a T_m_ value greater than 50 °C. The secondary structure of M15BT was predicted using The UNAFold Web Server, and the result is shown in Figure S3(B) (see Supplementary Information). Evidently, the 18-nt tail at the 3'-end of M15BT does not interfere with the formation of its secondary structure; thus, M15BT and M15B form the same hairpin-shaped secondary structure. This structure enables M15BT to be efficiently conjugated to tFNAs while retaining its specific binding capacity to the target (AML12 cells).

Finally, the changes in drug leakage rates of tFNA-encapsulated curcumin and ordinary curcumin aqueous solution were investigated to further evaluate the drug stability. As shown in Figure S5 (see Supplementary Information), the drug leakage rate of tFNA-encapsulated curcumin stored at 4 °C exhibited no statistically significant change (p > 0.05) within 14 days, with a mean value below 1%. In contrast, the drug leakage rate of the curcumin aqueous solution stored at 4 °C increased gradually, reaching 19.57 ± 1.596% on day 14. When stored at 25 °C, however, the drug leakage rates of both tFNA-encapsulated curcumin and curcumin aqueous solution increased progressively, reaching 72.38 ± 1.73% and 69.83 ± 4.532%, respectively. These results indicate that tFNA encapsulation significantly improves curcumin stability, but it also imposes strict requirements on storage temperature.

### 3.4. Mouse administration and efficacy against NAFLD

**Figure 5.**
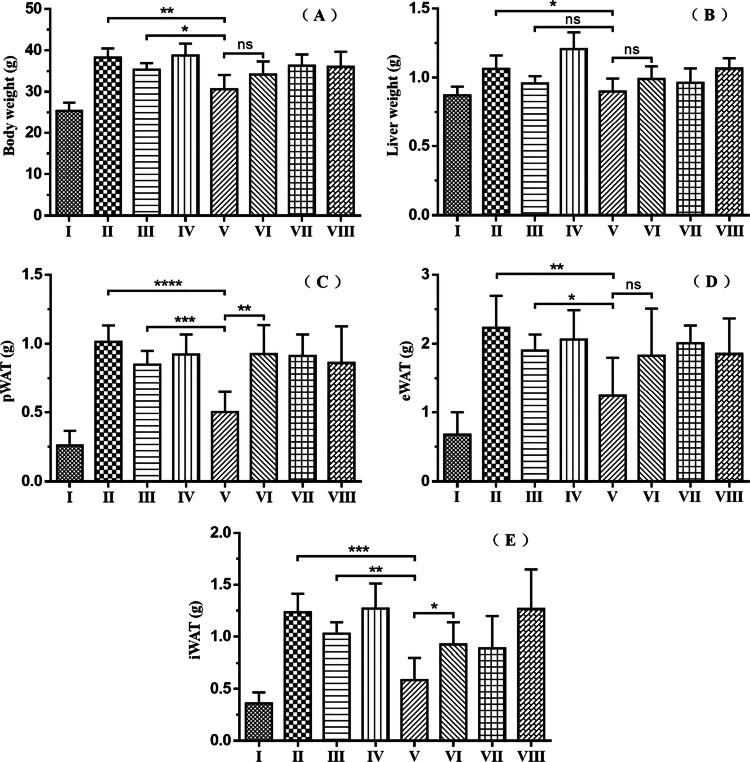
(A) Body weights of mice; (B) Liver weights of mice; (C) Perirenal fat weights of mice; (D) Epididymal fat weights of mice; (E) Inguinal fat weights of mice. *p < 0.05, **p < 0.01, ***p < 0.001, ****p < 0.0001; ns, not significant.

To evaluate the therapeutic efficacy of curcumin encapsulated by Targeted tFNAs on NAFLD, a high-fat diet (HFD)-induced NAFLD model was established by feeding mice a HFD. Subsequently, mice were grouped and administered treatments according to Table 1. As shown in Figure 5(A), compared to the High-fat Model group (II) (38.28 ± 2.21 g) and the Ordinary Curcumin group (III) (35.37 ± 1.563 g), the Curcumin Encapsulated by Targeted tFNAs group(V) (30.64 ± 3.439 g) demonstrated a remarkable ability to impede the weight gain and obesity triggered by HFD. Concurrently, Figure 5(B) shows a notable reduction in liver weight within Group V (0.899 ± 0.09464 g). Moreover, after the administration of curcumin encapsulated by Targeted tFNAs, the HFD-induced weight increments of the perirenal adipose tissue (pWAT) (0.503 ± 0.1458 g), epididymal adipose tissue (eWAT) (1.243 ± 0.5562 g), and inguinal adipose tissue (iWAT) (0.5855 ± 0.2137 g) were significantly mitigated, as illustrated in Figure 5(C),(D), and (E). Notably, significant differences were observed between Group III and Group V in body weight (Figure 5A), pWAT weight (Figure 5C: *p* = 0.0007), eWAT weight (Figure 5D: *p* = 0.0229), and iWAT weight (Figure 5E: *p* = 0.0011). These results confirm that curcumin encapsulated by Targeted tFNAs not only corrects overall metabolic abnormalities in HFD-induced mice but also exhibits superior therapeutic efficacy compared to ordinary curcumin. Additionally, a comparison between Group V and Group VI shows that targeted delivery is significantly superior to non-targeted delivery in reducing pWAT weight (Group V: 0.503 ± 0.1458 g; Group VI: 0.9265 ± 0.2094 g; *p* = 0.0023) and iWAT weight (Group V: 0.5855 ± 0.2137 g; Group VI: 0.924 ± 0.2147 g; *p* = 0.0209).

**Figure 6.**
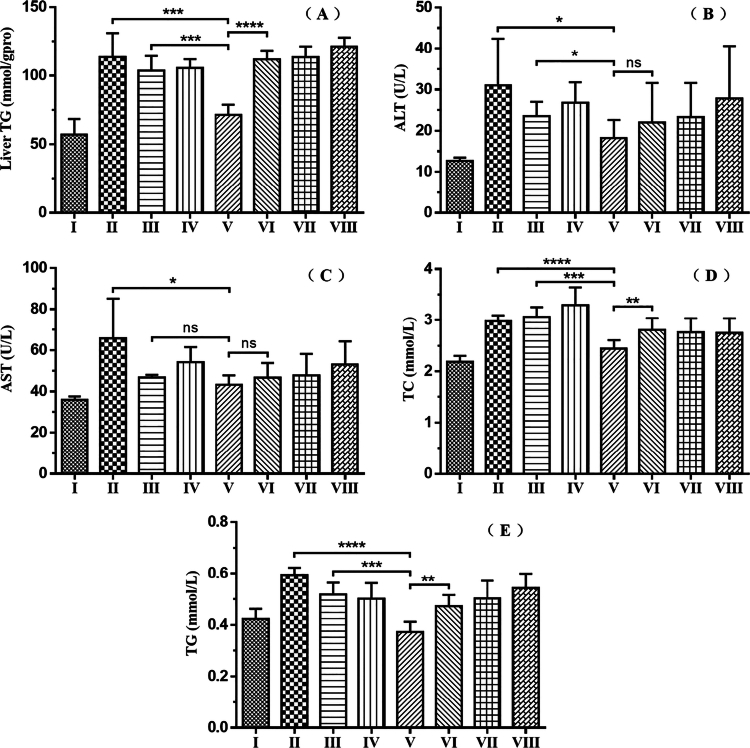
(A) Triglyceride (TG) content in mouse liver. (B) Alanine aminotransferase (ALT) activity in mouse plasma. (C) Aspartate aminotransferase (AST) activity in mouse plasma. (D) Total cholesterol (TC) content in mouse plasma. (E) Triglyceride (TG) content in mouse plasma. *p < 0.05, **p < 0.01, ***p < 0.001, ****p < 0.0001; ns, not significant.

Given that elevated triglyceride (TG) levels can induce fatty liver and elevated total cholesterol (TC) level can cause hyperlipidemia, the levels of TG and TC in mice were measured after different treatments. As shown in Figure 6(A),(D), and (E), curcumin encapsulated by Targeted tFNAs (Group V) (71.45 ± 7.411 mmol/gpro) exerted a substantial ameliorative effect (*p* = 0.0003) on the abnormal liver TG levels in HFD-induced mice (Group Ⅱ) (113.6 ± 17.48 mmol/gpro). Simultaneously, it significantly reduced plasma TG and TC levels in NAFLD mice (TG from 0.595 ± 0.02739 to 0.3733 ± 0.03882 mmol/L, *p* < 0.0001; TC from 2.98 ± 0.1108 to 2.443 ± 0.1654 mmol/L, *p* < 0.0001), effectively ameliorating dyslipidemia. A side-by-side comparison of the liver TG content (Group V: 71.45 ± 7.411 mmol/gpro; Group II: 113.6 ± 17.48 mmol/gpro; Group VI: 111.8 ± 6.548 mmol/gpro), plasma TG (Group V: 0.3733 ± 0.03882 mmol/L; Group II: 0.595 ± 0.02739 mmol/L; Group VI: 0.4733 ± 0.04274 mmol/L), and TC contents (Group V: 2.443 ± 0.1654 mmol/L; Group II: 2.98 ± 0.1108 mmol/L; Group VI: 2.81 ± 0.2298 mmol/L) of Group V with those of Group II and Group VI further validates that targeted delivery is not only markedly superior to the administration of ordinary curcumin (liver TG: *p* = 0.0003; plasma TG: *p* < 0.0001; plasma TC: *p* < 0.0001) but also far surpasses non–targeted delivery (liver TG: *p* < 0.0001; plasma TG: *p* = 0.0017; plasma TC: *p* = 0.0099).

As shown in Figure 6(B) and (C), curcumin encapsulated by Targeted tFNAs (Group V) was found to significantly curtail the plasma ALT (Group II: 31.03 ± 11.36 U/L; Group V: 18.22 ± 4.372 U/L; *p* = 0.0275) and AST (Group II: 65.77 ± 19.32 U/L; Group V: 43.32 ± 4.437 U/L; *p* = 0.0196) activities in NAFLD mice. These results confirm that curcumin encapsulated by Targeted tFNAs effectively alleviates lipid–mediated liver injury in NAFLD mice. Nevertheless, when it comes to reducing plasma ALT (Group V: 18.22 ± 4.372 U/L; Group VI: 22.03 ± 9.567 U/L; *p* = 0.395) and AST (Group V: 43.32 ± 4.437 U/L; Group VI: 46.68 ± 7.141 U/L; *p* = 0.3498) activities, no discernible difference was observed between targeted and untargeted delivery.

### 3.5. Liver-targeting validation of tFNAs

**Figure 7.**
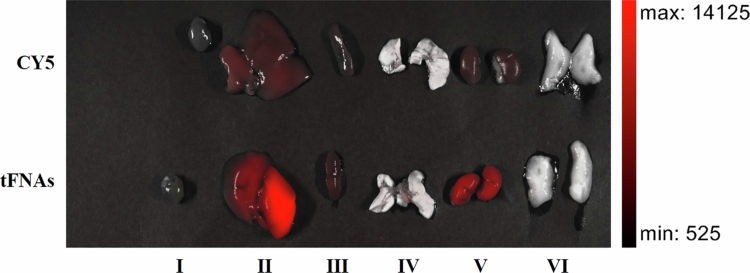
Photographs of mice internal organs. Ⅰ, Heart; Ⅱ, Liver; Ⅲ, Spleen; Ⅳ, Lung; Ⅴ, Kidney; Ⅵ, Groin fat.

To further verify the liver-targeted capability of targeted tFNAs, the CY5 fluorescent dye was used to label the TF1 and TF3 strands, generating CY5-labeled targeted tFNAs. One hour after tail vein administration, the mice were euthanized (in accordance with animal ethics protocols), and various internal organs were harvested; fluorescence images were captured, as shown in Figure 7. Compared to the CY5 aqueous solution (control group), CY5-labeled targeted tFNAs exhibited markedly stronger fluorescence intensity in the liver and kidneys. This confirms that targeted tFNAs accumulate effectively in the liver. The increased renal fluorescence may be attributed to the degradation of targeted tFNAs in the liver, which releases Cy5 into the bloodstream for renal excretion. This observation–the effective accumulation of targeted tFNAs in the liver–further explains why targeted tFNAs-encapsulated curcumin exhibits superior therapeutic efficacy in NAFLD treatment.

To further validate the targeting ability of targeted tFNAs, additional in vitro binding assays were conducted using hepatic and non-hepatic cell lines. RAW264.7 and GES-1 cells were used as non-hepatic controls, while AML12 cells served as the target. These cell lines were separately incubated with CY5-labeled targeted tFNAs; after three washes with phosphate-buffered saline (PBS), the fluorescence intensity was quantified via flow cytometry. Unexpectedly, the fluorescence intensity of AML12 cells (5.771 ± 0.1106) was significantly lower than that of the two non-hepatic cell lines (AML12 and RAW 264.7: *p* = 0.0004; AML12 and GES-1: *p* = 0.0078), with no significant difference observed between RAW 264.7 and GES-1 cells (RAW 264.7: 56.32 ± 7.830; GES-1: 41.92 ± 12.65; *p* = 0.1691, see Supplementary Information, Figure S6). This result indicates that targeted tFNAs can distinguish AML12 cells from non-hepatic cell lines, but it contradicts the earlier aptamer binding results (Figure 2)—why was the fluorescence intensity of target cells (AML 12) lower than that of non-target cells? To address this discrepancy, we performed microscopic imaging of cells treated with CY5-labeled targeted tFNAs. As shown in Figure S7, CY5-labeled targeted tFNAs did not exhibit the distinct intracellular punctate staining effect typical of other fluorescent dyes; instead, they produced a broad area of faint red fluorescence in the field of view. We hypothesize that this phenomenon arises from lysosomal engulfment: after entering cells, targeted tFNAs are internalized into lysosomes, where acidic conditions or lysosomal enzymes induce fluorescence quenching (a reduction in fluorescence intensity due to environmental interference). Notably, targeted tFNAs are more readily phagocytosed by AML12 cells than by non-hepatic cells, which accounts for their lower fluorescence intensity. Thus, the targeting mechanism of this delivery system may involve not only surface binding to hepatic cells but also active internalization into hepatocytes-facilitating the delivery of therapeutic agents directly into the target cells.

## 4. Discussion

DNA tetrahedrons are rigid nanocages self-assembled from four single-stranded DNA molecules via complementary base pairing. Their interior contains a hydrophobic cavity formed by the hydrophobic bases of DNA double helices (such as purines and pyrimidines), which provides an ideal environment for accommodating hydrophobic molecules. Curcumin, a highly hydrophobic polyphenolic molecule (logP ≈ 3.3), tends to aggregate and precipitate in water. When mixed with DNA tetrahedrons, curcumin spontaneously embeds into the hydrophobic cavity of the tetrahedron through hydrophobic interactions, forming an encapsulation structure similar to a “molecular pocket.” This reduces the contact area between curcumin and water, leading to a decrease in the system's free energy (ΔG < 0). Additionally, the phenolate anions of curcumin may interact with locally positively charged regions of DNA bases (e.g. the N7 site of guanine) through weak electrostatic attraction, aiding its localization at the cavity entrance.

Moreover, the rigid cavity of the DNA tetrahedron isolates curcumin from the external environment (water, oxygen, light), forming a physical barrier. Additionally, encapsulated curcumin is restricted from free rotation or vibration by spatial hindrance, reducing its degradation caused by photoisomerization or oxidation. Furthermore, the cavity limits the contact between multiple curcumin molecules, effectively inhibiting their hydrophobic aggregation and precipitation.

In summary, the encapsulation of curcumin by DNA tetrahedrons essentially relies on the spatial embedding within the hydrophobic cavity, supplemented by weak electrostatic adsorption for auxiliary positioning. This encapsulation significantly reduces curcumin’s hydrolysis, oxidation, and photodegradation rates through physical isolation (from water/oxygen) and spatial confinement (restricting molecular motion). Concurrently, the negatively charged hydrophilic phosphate backbones on the tFNAs surface form a hydration shell in aqueous solutions, endowing the curcumin-tFNAs complex with good water solubility (solubility > 50 μg/mL, vs. < 5 μg/mL for free curcumin; see Section 3.3). This mechanism provides an important paradigm for the design of DNA–based nanocarriers for other hydrophobic drugs.

Herein, the selected aptamer M15B exhibits recognition specificity for AML12 cells. The incorporation of M15BT (prepared based on aptamer M15B) onto tetrahedral framework nucleic acids (tFNAs) not only enables hepatocyte-targeting but may also stabilize the tFNAs structures. Encapsulation within targeted tFNAs significantly enhances curcumin’s bioavailability, stabilizing the compound in blood, facilitating efficient hepatic delivery, and achieving final targeted accumulation in the liver. As a result, curcumin administered at a concentration as low as 50 μg/mL and a dosage volume of 10 μL/g body weight achieved remarkable therapeutic efficacy against NAFLD. Compared with a previous study reporting intravenous administration of free curcumin at 10 mg/kg body weight (Hegde et al. 2023), the dosage of targeted tFNAs-encapsulated curcumin in the present study (0.5 mg/kg body weight) was significantly reduced (20-fold lower). This undoubtedly corroborates the high efficiency of the targeted delivery approach developed in this research.

In terms of therapeutic efficacy, curcumin encapsulated in targeted tFNAs not only improved global metabolic abnormalities in high-fat diet (HFD)-fed mice but also demonstrated significantly superior therapeutic performance compared to ordinary curcumin. Key metrics-including body weight, liver weight, perirenal white adipose tissue (pWAT), epididymal white adipose tissue (eWAT), inguinal white adipose tissue (iWAT), liver triglyceride (TG) levels, plasma total cholesterol (TC), and plasma TG-were all significantly reduced or improved in the targeted administration group compared with the High-fat Model group.

It is a well-established fact that alanine aminotransferase (ALT) and aspartate aminotransferase (AST) are pivotal enzymes in mammalian protein metabolism, expressed in various tissues and organs with the highest concentrations in hepatocyte cytoplasm. When tissues and organs in the body are damaged or affected by disease, a portion of ALT and AST is released into the bloodstream, leading to an increase in their activity in plasma. Consequently, in liver-function assessments, the plasma ALT activity is customarily employed as an index of damaged liver cells, while the plasma AST activity serves as an indicator of hepatocyte necrosis. Although targeted tFNA-encapsulated curcumin effectively alleviated lipid-mediated liver injury in NAFLD mice, no significant differences were observed between targeted and non–targeted administrations in reducing plasma alanine transaminase (ALT) and aspartate transaminase (AST) activities. We hypothesize that the elevated plasma ALT and AST activities in NAFLD mice may not be solely attributable to hepatocyte damage but could potentially be precipitated by the damage or affliction of other cell types.

## 5. Conclusions

To address the poor aqueous solubility of curcumin and its lack of liver targeting (key limitations for NAFLD therapy), this study developed tetrahedral framework nucleic acids (tFNAs) as a nanocarrier for curcumin. Through conjugation with liver-specific aptamers, an aptamer-mediated liver-targeted curcumin delivery system was constructed for NAFLD therapy.

Initially, the aptamer M15B was screened via SELEX method. This aptamer exhibits highly specific recognition for mouse hepatocytes (AML12 cells). Subsequently, through the rational design of four ssDNAs, successful tFNAs assembly and curcumin encapsulation were achieved. This strategy significantly improved curcumin’s aqueous solubility (>50 μg/mL, vs. <5 μg/mL for free curcumin) and stability (drug leakage rate <1% at 4 °C for 14 days). Finally, tFNAs were conjugated with the aptamer M15B to prepare targeted tFNAs, completing the fabrication of the liver–targeted curcumin formulation.

Following liver-targeted delivery, the therapeutic effect of curcumin on NAFLD was markedly enhanced. Multiple key parameters—including mouse body weight, perirenal/epididymal/inguinal white adipose tissue (pWAT/eWAT/iWAT) weight, hepatic triglyceride (TG) levels, and plasma total cholesterol (TC) levels—demonstrated that targeted tFNAs-encapsulated curcumin exhibited significantly higher efficacy in NAFLD treatment than ordinary curcumin. Additionally, the fluorescence labeling experiment further confirmed the hepatic targeting capability of tFNAs.

This study successfully establishes a tFNAs-based aptamer-mediated liver-targeted delivery platform. This platform not only improves curcumin’s clinical applicability for NAFLD but also provides a new therapeutic option for this disease. More importantly, the platform is not limited to curcumin; it can be extended to the liver-targeted delivery of other hydrophobic drugs (e.g. resveratrol, quercetin), holding great potential for broad applications in hepatic disease therapy.

## Acknowledgements

The authors thank financially support from the Basic Public Welfare Research Program of Zhejiang Province (No. LTGY23H290002), National Natural Science Foundation of China (No. 81603254; No. 82374102), Leading Talents + X Program of Zhejiang Province (No. 2025C02174).

## Author contributions

Conceptualization: S.C., Y.H., X.D.; data curation: S.C., Y.L., L.C., L.Z., J.W.; formal analysis: S.C., Y.L., S.M.; investigation: S.C., Y.L., S.M.; methodology: S.C., Y.L., S.M.; validation: S.M.; visualization: L.C., L.Z., Z.Y.; writing-original draft: S.C., Y.L., S.M.; writing-review & editing: S.C., Y.H.; resources: S.C., Y.H., X.D.; funding acquisition: S.C., X.D.; project administration: S.C., X.D.; all authors agree to be accountable for all aspects of the work.

## Supplementary Material

Supplementary Material Aptamer-mediated liver-targeted curcumin delivery system based on tetrahedral framework nucleic acids for NAFLD

Supplementary Material Figure S4 TEM photographs of untargeted tFNAs:

## Data Availability

All details of experimental data are presented in the article or additional file.
