# Supplementary material for: Aptamer-mediated liver-targeted curcumin delivery system based on tetrahedral framework nucleic acids for NAFLD
Source: Drug Deliv. 2025 Nov 9;32(1):2576222. doi: 10.1080/10717544.2025.2576222 (PMC12604106; doi:10.1080/10717544.2025.2576222)
Supplement: Supplementary Material — Aptamer-mediated liver-targeted curcumin delivery system based on tetrahedral framework nucleic acids for NAFLD [file IDRD_A_2576222_SM3786.docx]

**Supplementary information**

**Aptamer-mediated liver-targeted curcumin delivery system based on tetrahedral framework nucleic acids for NAFLD**

***Preparation of ssDNAs Library:***

The sequence of Library (L1): CACGGATCCTGACAAG-N40-CAGCTCCGTCCG. Here, N40 represents a stretch of 40 random nucleotides, which endows the library with the necessary diversity for subsequent screening processes.

Forward Primer (FP): CACGGATCCTGACAAG. Reverse Primer 1 (RP1): CGGACGGAGCTG.

Reverse Primer 2 (RP2): A20-Sp-CGGACGGAGCTG, where "Sp" denotes a spacer, and A20 represents 20 Adenosines.

For the initial library preparation, the L1 sequence served as the template. And for all subsequent library preparation, the ssDNAs obtained from previous screening rounds were utilized as templates. The first-step PCR amplification was executed using FP and RP1 as primers. This reaction yielded the double-stranded DNA product designated as LFP. Subsequently, taking LFP as the template, a second-round PCR amplification was carried out with FP and RP2 as primers, resulting in the generation of the double-stranded DNA product LPD. The shorter-chain ssDNAs were isolated with denaturing PAGE. These separated ssDNAs were then collected and constituted the ssDNAs library, which served as the starting material for the aptamer selection process.

***Selection of Nucleic Acid Aptamers:***

The ssDNAs library was incubated with the positive target (mouse hepatocytes AML12) at 37 ℃ for 1 hour. Following incubation, the sample was centrifuged at 4000 r/min for 5 minutes. The supernatant, which contains unbound ssDNAs and other soluble components, was carefully discarded. The remaining cell-bound ssDNAs in the precipitate were then washed twice with PBS solution to remove any non-specifically adsorbed contaminants. Subsequently, 400 μL NaOH (20 mmol/L) was added to the washed precipitate. This alkaline solution facilitated the dissociation of the ssDNAs from the target cells. The dissociation process was carried out in a metal bath maintained at 37 ℃ for 3 minutes. After dissociation, 4 μL HCl (2 mol/L) was added to neutralize the alkaline environment. The sample was then centrifuged again at 4000 r/min for 5 minutes, and the supernatant, now containing the dissociated ssDNAs, was collected in a 1.5-mL centrifuge tube. The collected supernatant was denatured at 95 ℃ for 3 minutes and then rapidly frozen at -20 ℃ for 1 minute. Using these ssDNAs as templates, a new ssDNAs library was constructed for the subsequent negative-screening step.

The ssDNAs library obtained from the positive-screening process was then incubated with the negative target, mouse mononuclear macrophages RAW264.7, at 37 ℃ for 1 hour. After incubation, the sample was centrifuged at 4000 r/min for 5 minutes, and the supernatant was collected in a 1.5-mL centrifuge tube. This supernatant, containing ssDNAs that did not bind to the negative target, was denatured at 95 ℃ for 3 minutes and frozen at -20 ℃ for 1 minute. Using these ssDNAs as templates, a new library was prepared for the next round of screening.

After 10 rounds of the overall screening process, the ssDNAs obtained every 5 rounds of screening were subjected to sequencing analysis. The sequencing primers employed were FP and RP1. Based on the sequencing results, the aptamers of interest were labeled with the green fluorescent group, 6-carboxyfluorescein (FAM), to create fluorescent probes. In the subsequent binding-specificity analysis, RAW264.7 cells were designated as the negative target, and AML12 cells were used as the positive target. After incubating the fluorescent probes with the target cells, the unbound probes were removed through a series of washing steps. The fluorescence signal of the remaining bound probes was then detected using flow cytometry. The binding specificity of the aptamer was determined by comparing the fluorescence signals obtained from the positive and negative targets. A significant difference in fluorescence signals between the two targets indicated a high-specificity binding of the aptamer to the positive target. RAW 264.7 (CL-0190) and AML12 (CL-0602) cells were purchased from Pricella Biotechnology Co., Ltd.

***Design of ssDNAs:***

**TF1**: ATTTATCACCCGCCATAGTAGACGTATCACCAGGCAGTTGAGACGAACATTCCTAA-

GTCTGAA;

**TF2**: ACATGCGAGGGTCCAATACCGACGATTACAGCTTGCTACACGATTCAGACTTAGGA-

ATGTTCG;

**TF3**: ACTACTATGGCGGGTGATAAAACGTGTAGCAAGCTGTAATCGACGGGAAGAGCAT-

GCCCATCC;

**TF4A**: ACGGTATTGGACCCTCGCATGACTCAACTGCCTGGTGATACGAGGATGGGCATG-

CTCTTCCCG;

**TF4B**: ACGGTATTGGACCCTCGCATGACTCAACTGCCTGGTGATACGAGGATGGGCATG-

CTCTTCCCGAAAAAAAACCAAACCAAACCAAACG;

**M15B**: CACGGATCCTGACAAGGGGGGGGGCCCCTCTTTTGTTCTTGTTTCCCTCCCCC-

TTACAGCTCCGTCCG. **M15B** is the aptamer screened in this study.

**M15BF**: CACGGATCCTGACAAGGGGGGGGGCCCCTCTTTTGTTCTTGTTTCCCTCCCCC-

TTACAGCTCCGTCCG-FAM. **M15BF** is the aptamer **M15B** with a FAM fluorescent label.

**TF1CY5**: CY5-ATTTATCACCCGCCATAGTAGACGTATCACCAGGCAGTTGAGACGAACAT-

TCCTAAGTCTGAA; **TF1CY5** is **TF1** with a **CY5** fluorescent label.

**TF3CY5**: CY5-ACTACTATGGCGGGTGATAAAACGTGTAGCAAGCTGTAATCGACGGGAA-

GAGCATGCCCATCC; **TF3CY5** is **TF3** with a **CY5** fluorescent label.

**M15BT**: CACGGATCCTGACAAGGGGGGGGGCCCCTCTTTTGTTCTTGTTTCCCTCCCCC-

TTACAGCTCCGTCCGTTTGGTTTGGTTTGGTTT. **M15BT** was designed based on **M15B** and **TF4B**.

***Detection of Alanine Aminotransferase (ALT) in Plasma:***

Once blood collection was completed, pre-chilled the centrifuge to 4 ℃. Then, subjected the blood sample to centrifugation at 3000 r/min for 10 minutes. Gently aspirated the upper-layer serum and transferred it into a fresh 1.5-mL centrifuge tube for subsequent use. Adhered to the instructions provided with the Alanine Aminotransferase Assay Kit (Nanjing Jiancheng Bioengineering Institute, China, C009-2-1). Pipetted 5 μL of mouse plasma into a well of a 96-well plate. Subsequently, added 20 μL of the ALT substrate solution. Incubated the plate at 37 ℃ for 30 minutes to allow the enzymatic reaction to proceed. After the initial incubation, introduced 20 μL of 2, 4-dinitrophenylhydrazine solution into each well. Returned the plate to the 37 ℃ incubator for another 30-minute reaction period. Following this, added 200 μL of 0.4 mmol/L NaOH to each well. Gently shook the plate horizontally to ensure thorough mixing. Let the plate stand at room temperature for 15 minutes. Finally, measured the absorbance at a wavelength of 510 nm using a microplate reader. Calculated the ALT activity by referring to the pre-established standard curve.

***Detection of Aspartate Aminotransferase (AST) in Plasma:***

Once blood collection was completed, pre-cooled the centrifuge to 4 ℃. Centrifuged the blood sample at 3000 r/min for 10 minutes. Carefully extracted the upper-layer plasma and transferred it to a new 1.5mL centrifuge tube for future analysis. Followed the protocol of the Aspartate Aminotransferase Assay Kit (Nanjing Jiancheng Bioengineering Institute, Nanjing, China, C010-2-1). Transferred 5 μL of mouse plasma into a well of a 96-well plate. Added 20 μL of the AST substrate solution to the well. Incubated the plate at 37 ℃ for 30 minutes to facilitate the reaction. After the first incubation, added 20 μL of 2, 4-dinitrophenylhydrazine solution to each well. Placed the plate back in the 37 ℃ incubator for an additional 30-minute reaction. Subsequently, introduced 200 μL of 0.4 mmol/L NaOH into each well. Shaked the plate horizontally to ensure homogeneous mixing. Allowed the plate to rest at room temperature for 15 minutes. Measured the absorbance at a wavelength of 510 nm using a microplate reader. Determined the AST activity based on the standard curve.

***Detection of Triglyceride (TG) in Plasma:***

Referred to the instructions of the Tissue Triglyceride Content Assay Kit (Applygen, Beijing, China, E1003). Diluted the standard solution and set up a control reaction tube with a 0-concentration standard. Transferred 30 μL of plasma and each dilution of the standard solution into separate wells of a 96-well plate. Added 170 μL of the pre-prepared working solution (prepared by mixing R1 and R2 in a 4:1 ratio) to each well. Incubated the plate at 37 ℃ for 15 minutes to enable the reaction. After the incubation period, measured the absorbance at a wavelength of 550 nm using a microplate reader. Plotted the standard curve based on the absorbance values of the standard dilutions and calculated the TG concentration in the plasma samples.

***Detection of Total Cholesterol (TC) in Plasma:***

Followed the guidelines provided with the Tissue Total Cholesterol Content Assay Kit (Applygen, Beijing, China, E1005). Diluted the standard cholesterol solution and set up a control tube with a 0-concentration standard. Pipetted 30 μL of plasma and each dilution of the standard solution into individual wells of a 96-well plate. Added 170 μL of the working solution (prepared by mixing R1 and R2 in a 4:1 ratio) to each well. Incubated the plate at 37 ℃ for 15 minutes to allow the reaction to occur. After incubation, measured the absorbance at a wavelength of 550 nm using a microplate reader. Constructed the standard curve using the absorbance data from the standard dilutions and calculated the TC concentration in the plasma samples.

***Detection of TG in Liver:***

Adhered to the instructions of the Tissue Triglyceride Content Assay Kit (Applygen, Beijing, China, E1003). Diluted the standard TG solution and set up a control tube with a 0-concentration standard. Accurately weighed 20 mg of liver tissue and placed it in a sterile 1.5-mL centrifuge tube. Added the lysis buffer at a ratio of 20 μL per mg of tissue. Disrupted the liver tissue using a fully-automated rapid tissue grinder. After thorough lysis for 10 minutes, heated the tube in a metal bath at 70 ℃ for 10 minutes. Centrifuged the tube at 2000 r/min at room temperature for 5 minutes. Carefully collected the supernatant. Transferred 10 μL of the supernatant and each dilution of the standard solution into separate wells of a 96-well plate. Added 190 μL of the working solution (prepared by mixing R1 and R2 in a 4:1 ratio) to each well. Incubated the plate at 37 ℃ for 15 minutes. After incubation, measured the absorbance at a wavelength of 550 nm using a microplate reader. Plotted the standard curve based on the standard dilutions and calculated the TG concentration in the liver tissue.

***Fluorescence Imaging of Animal Tissues:***

Substituted TF1 with TF1CY5 and TF3 with TF3CY5 to prepare CY5-labeled targeted tFNAs. Randomly allocated six male C57BL/6 mice (weighing 20-25 g) into two groups. Administered CY5 (2 μmol/L) and CY5-labeled targeted tFNAs via intravenous (i.v.) injection into the tail vein of the mice in each group respectively. The dosage was calculated as 10 μL of the solution per gram of the mouse's body weight. One hour after injection, anesthetized the mice and then sacrificed them. Immediately after sacrifice, collected the heart, liver, spleen, lungs, kidneys, and subcutaneous fat tissues. Placed the collected tissue samples in the Tanon fully-automated multi-functional imaging system to capture fluorescence images.


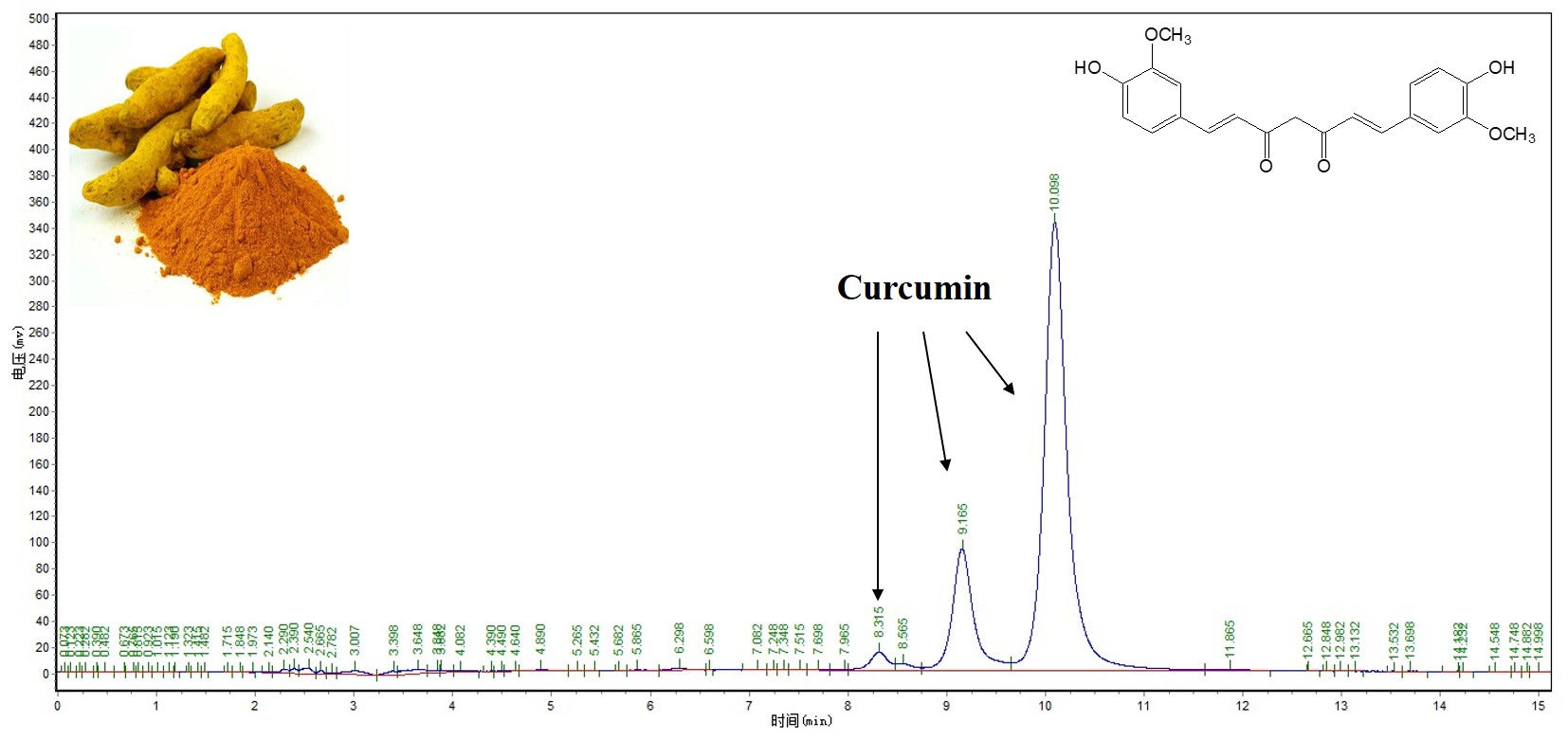


**Figure S1.** HPLC spectrum of curcumin.
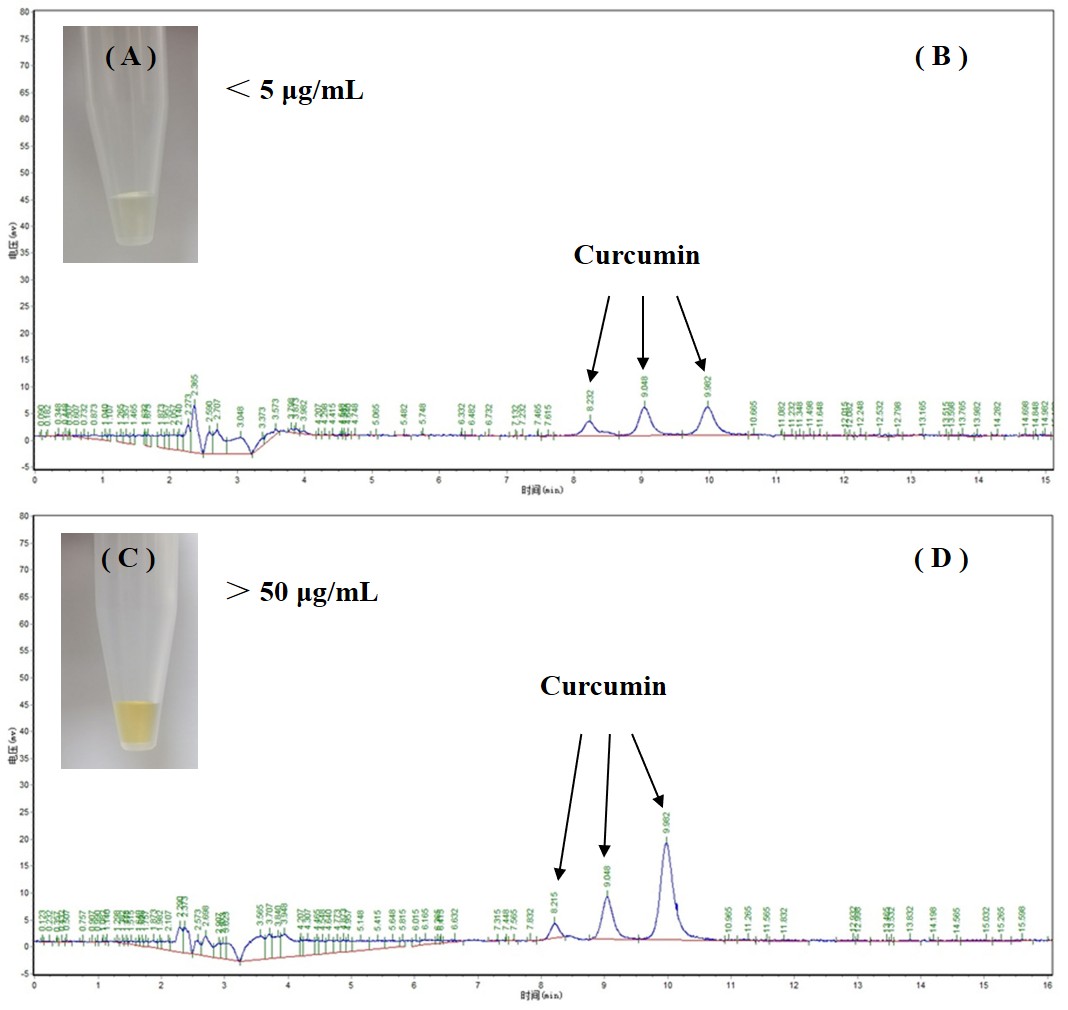


**Figure S2.** Curcumin in solutions and HPLC spectrum. A) Curcumin aqueous solution. B) HPLC spectrum of (A) solution diluted by 9 times methanol. C) Aqueous solution of curcumin encapsulated by tFNAs. D) HPLC spectrum of (C) solution diluted by 9 times methanol.


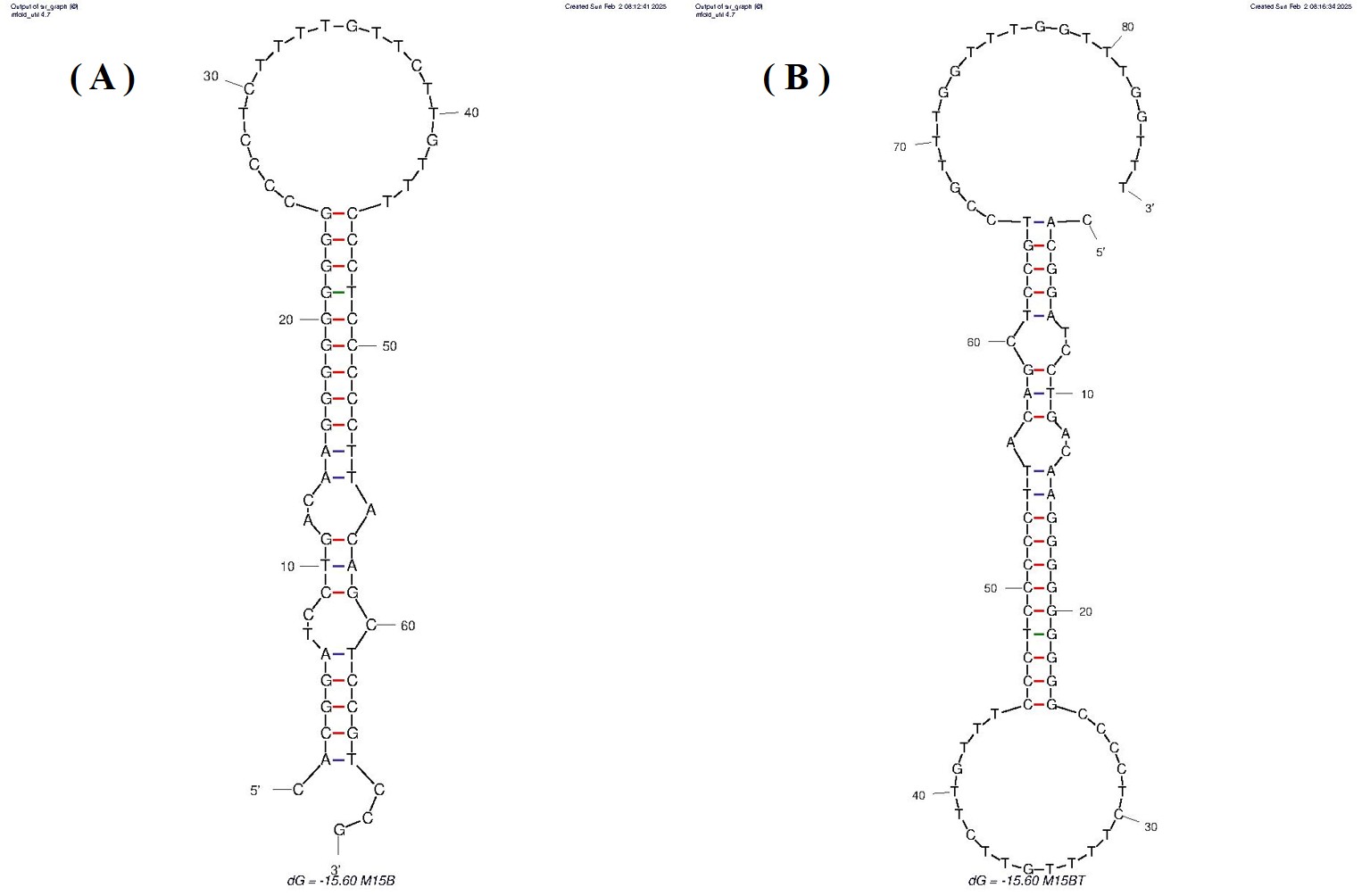


**Figure S3.** A) The secondary structure of M15B. B) The secondary structure of M15BT.


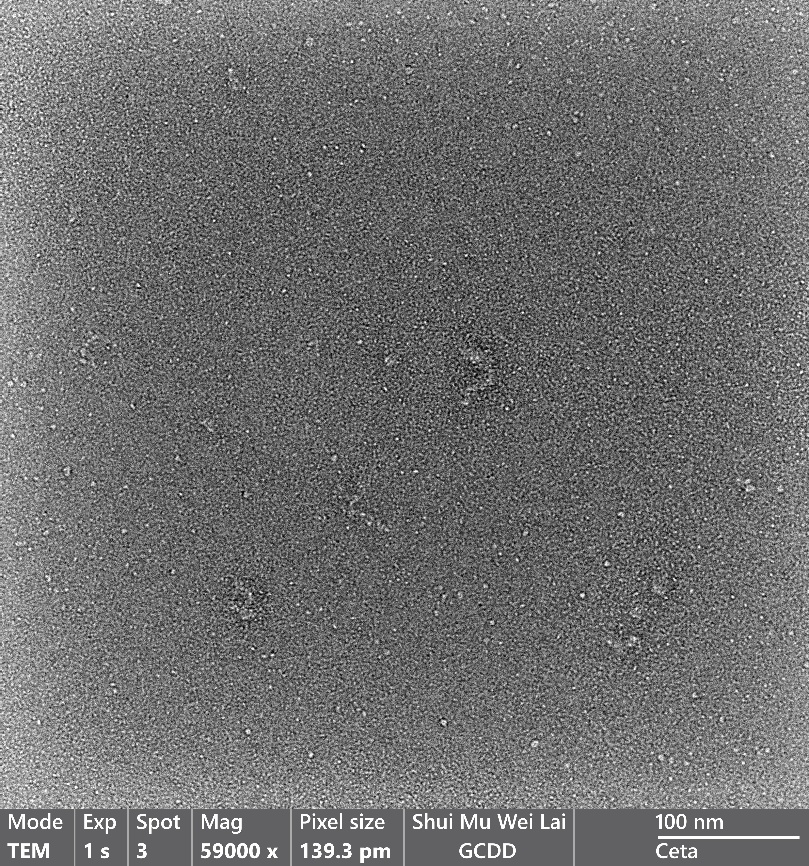


**Figure S4.** TEM photographs of untargeted tFNAs.


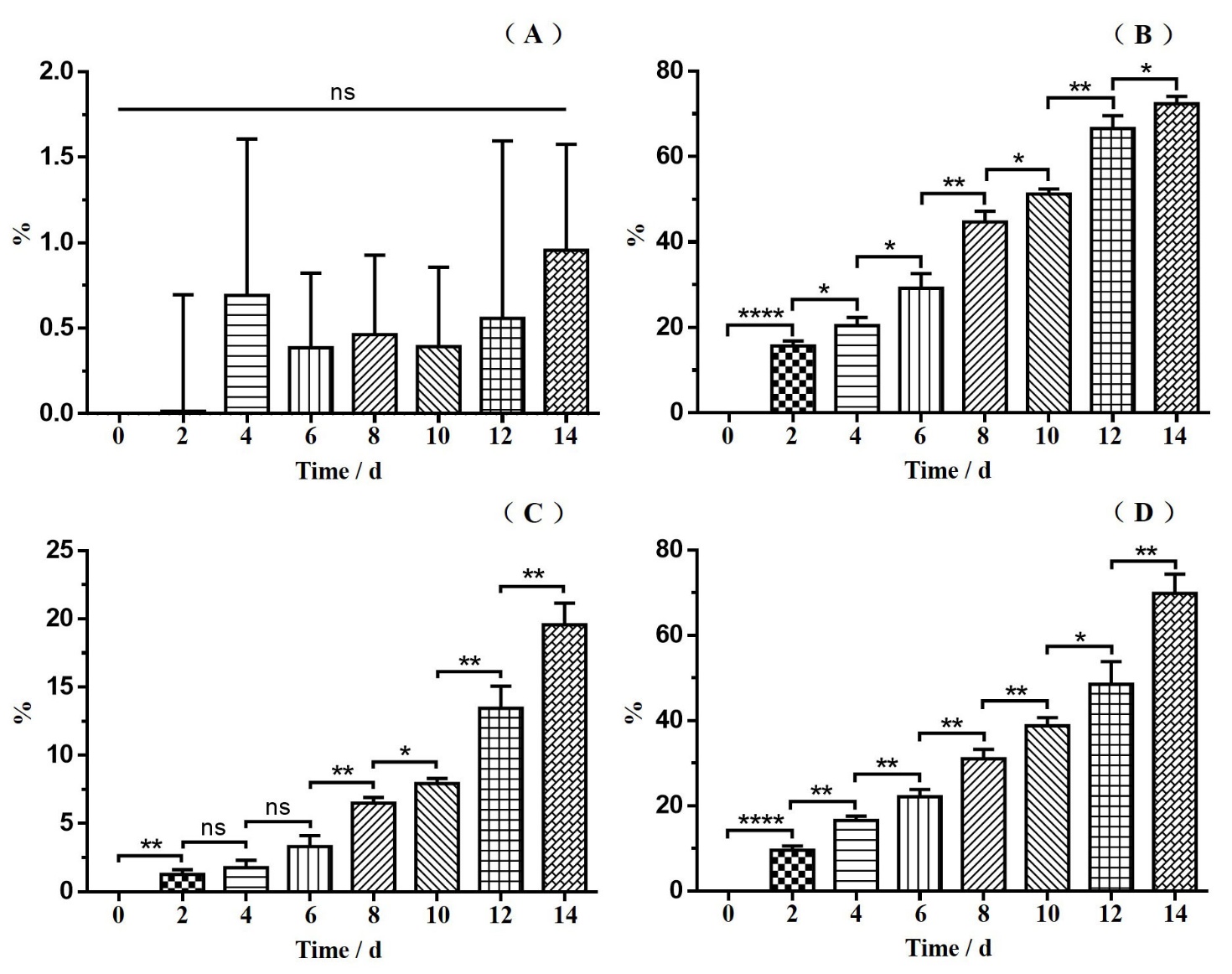


**Figure S5.** Drug leakage rates over a 14-day period. A) Aqueous solution of curcumin encapsulated by targeted tFNAs at 4 ℃. B) Aqueous solution of curcumin encapsulated by targeted tFNAs at 25 ℃. C) Curcumin aqueous solution at 4 ℃. D) Curcumin aqueous solution at 25 ℃.


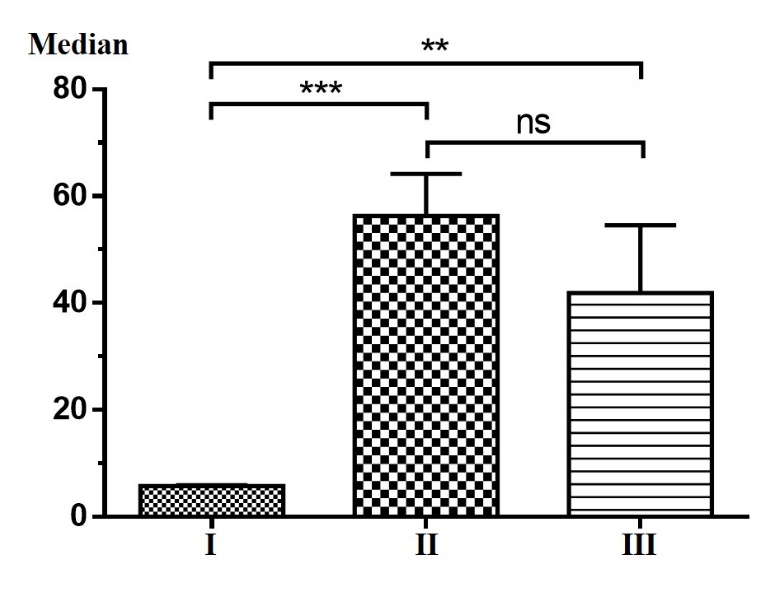


**Figure S6.** The median fluorescence intensity of cells after administration of CY5-labeled targeted tFNAs. Ⅰ) AML12. Ⅱ) RAW264.7. Ⅲ) GES-1.


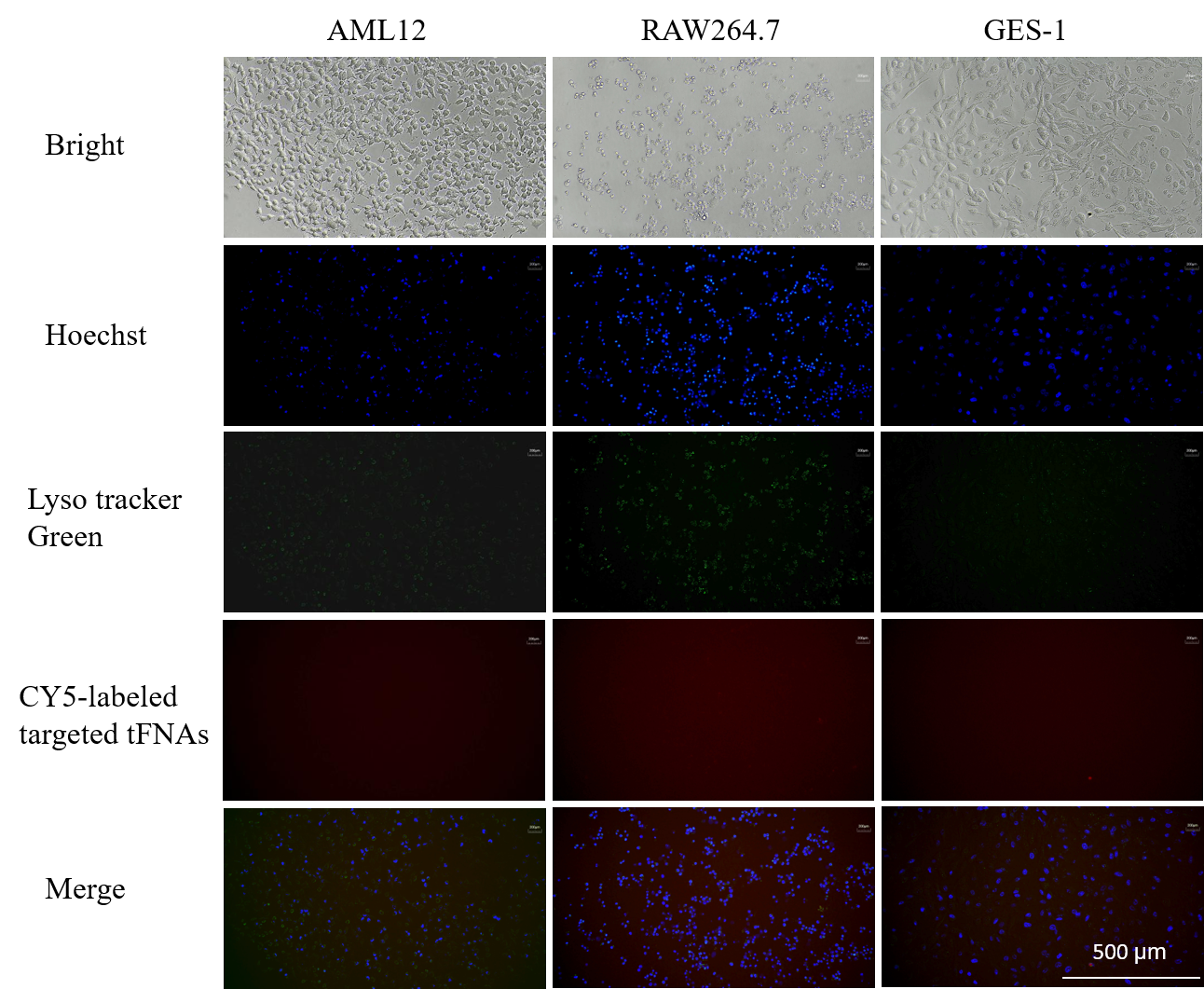


**Figure S7.** Microscopic imaging of cells after administration of CY5-labeled targeted tFNAs.
