# Supplementary material for: Aptamer-mediated liver-targeted curcumin delivery system based on tetrahedral framework nucleic acids for NAFLD
Source: Drug Deliv. 2025 Nov 9;32(1):2576222. doi: 10.1080/10717544.2025.2576222 (PMC12604106; doi:10.1080/10717544.2025.2576222)

**Figure 3 (A) PAGE electrophoretogram of the targeted tFNAs:**

**Figure 3 (B) PAGE electrophoretogram of the untargeted tFNAs:**


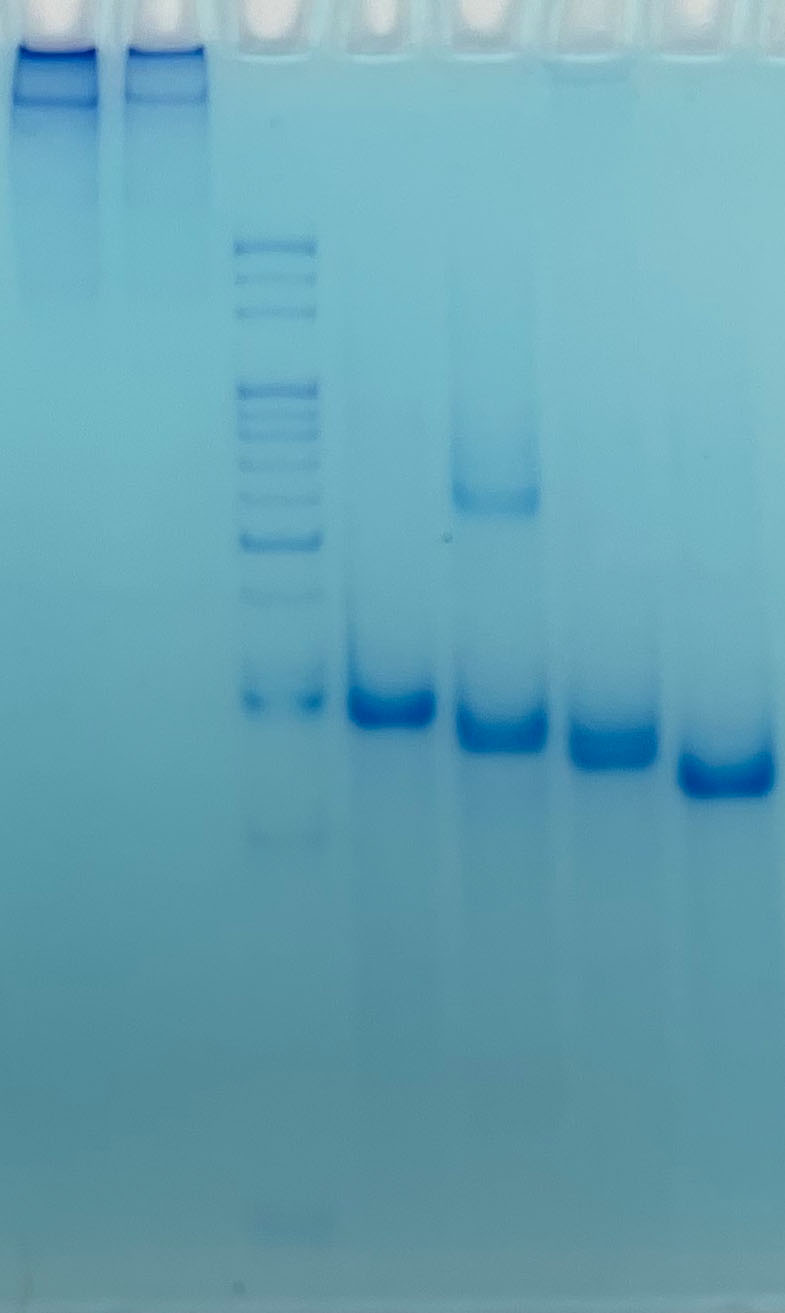


**Figure 4 (A) TEM photographs of ssDNAs (TF1):**


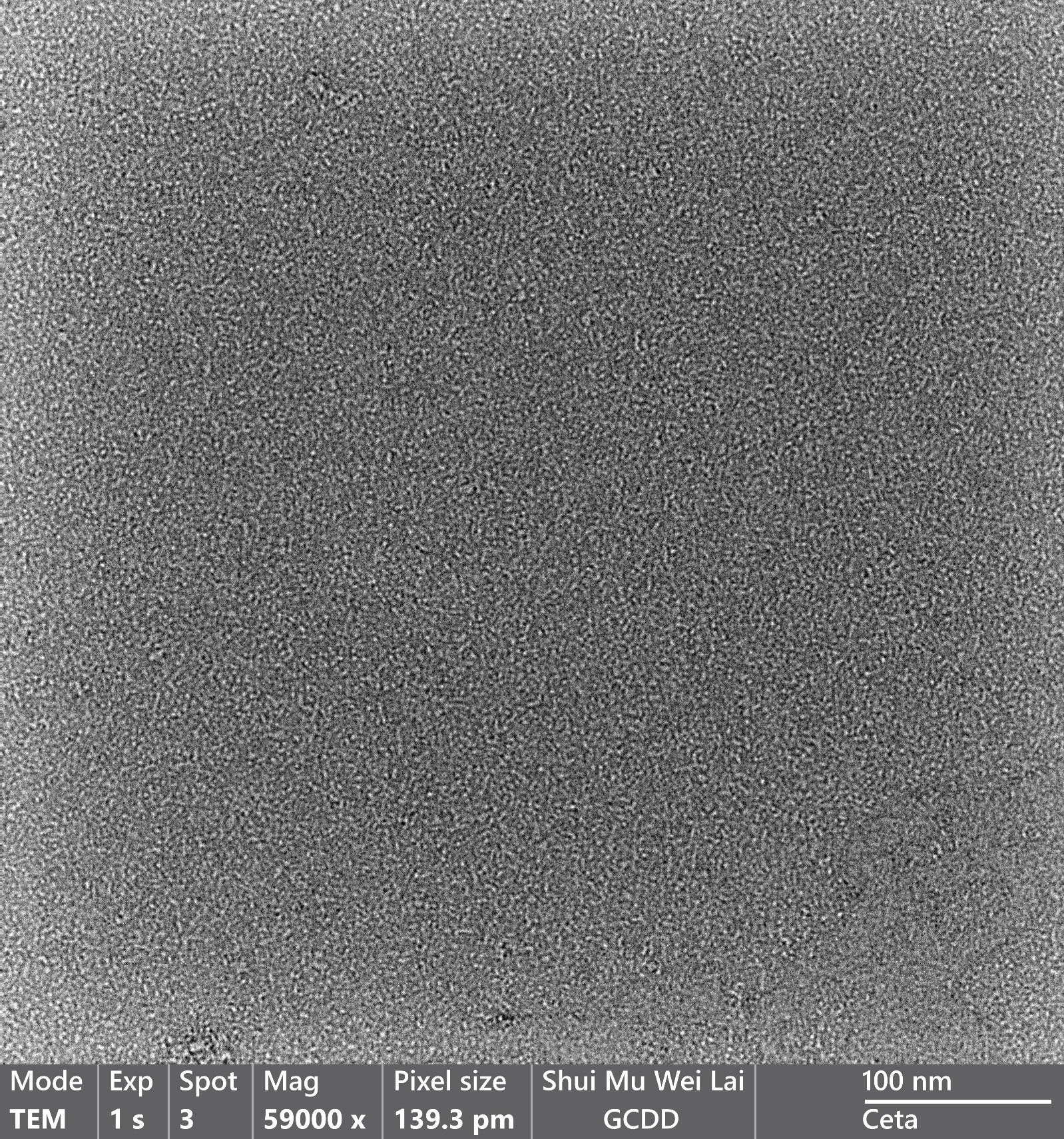


**Figure 4 (B) TEM photographs of targeted tFNAs:**


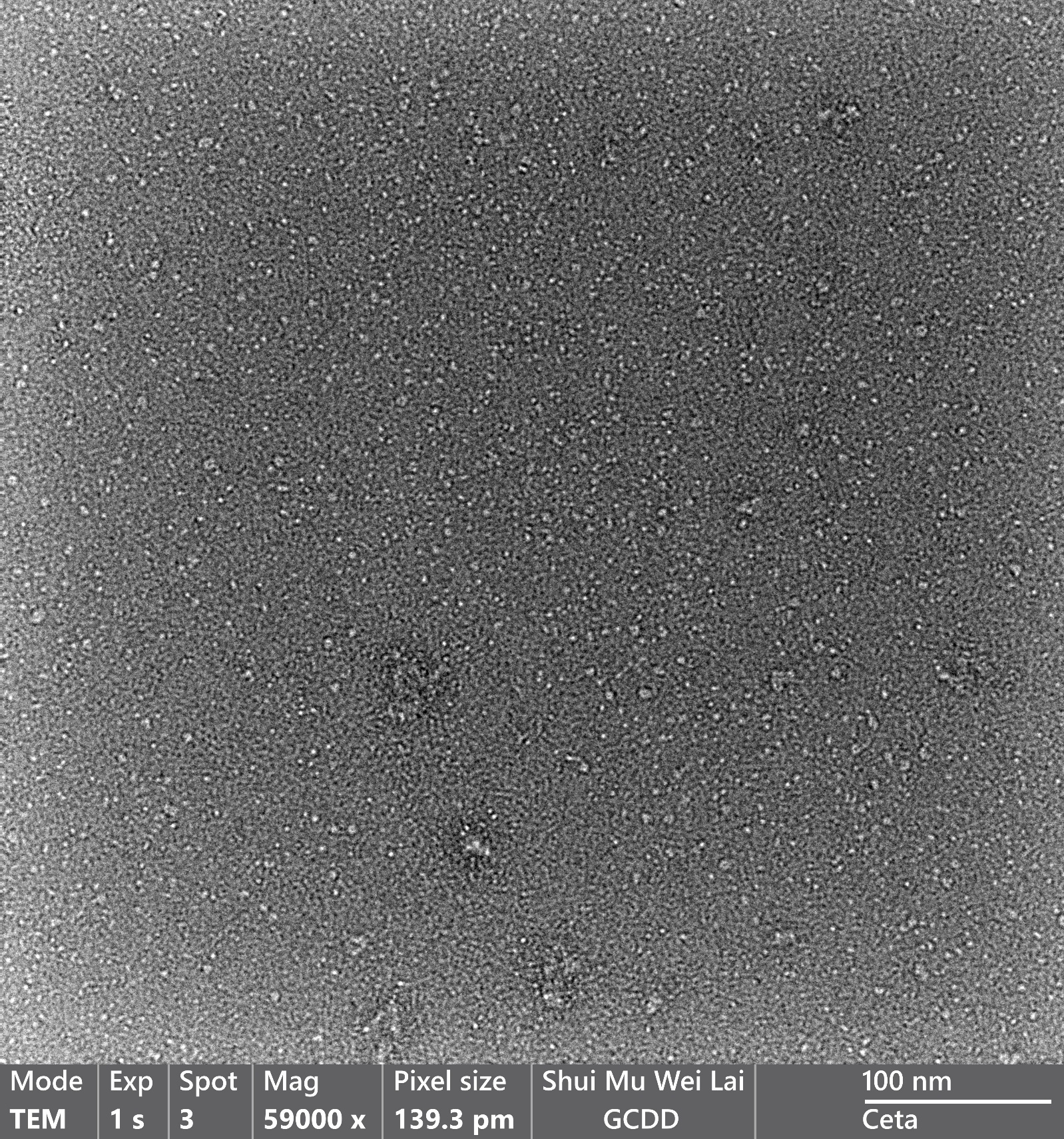


**Figure S4 TEM photographs of untargeted tFNAs:**


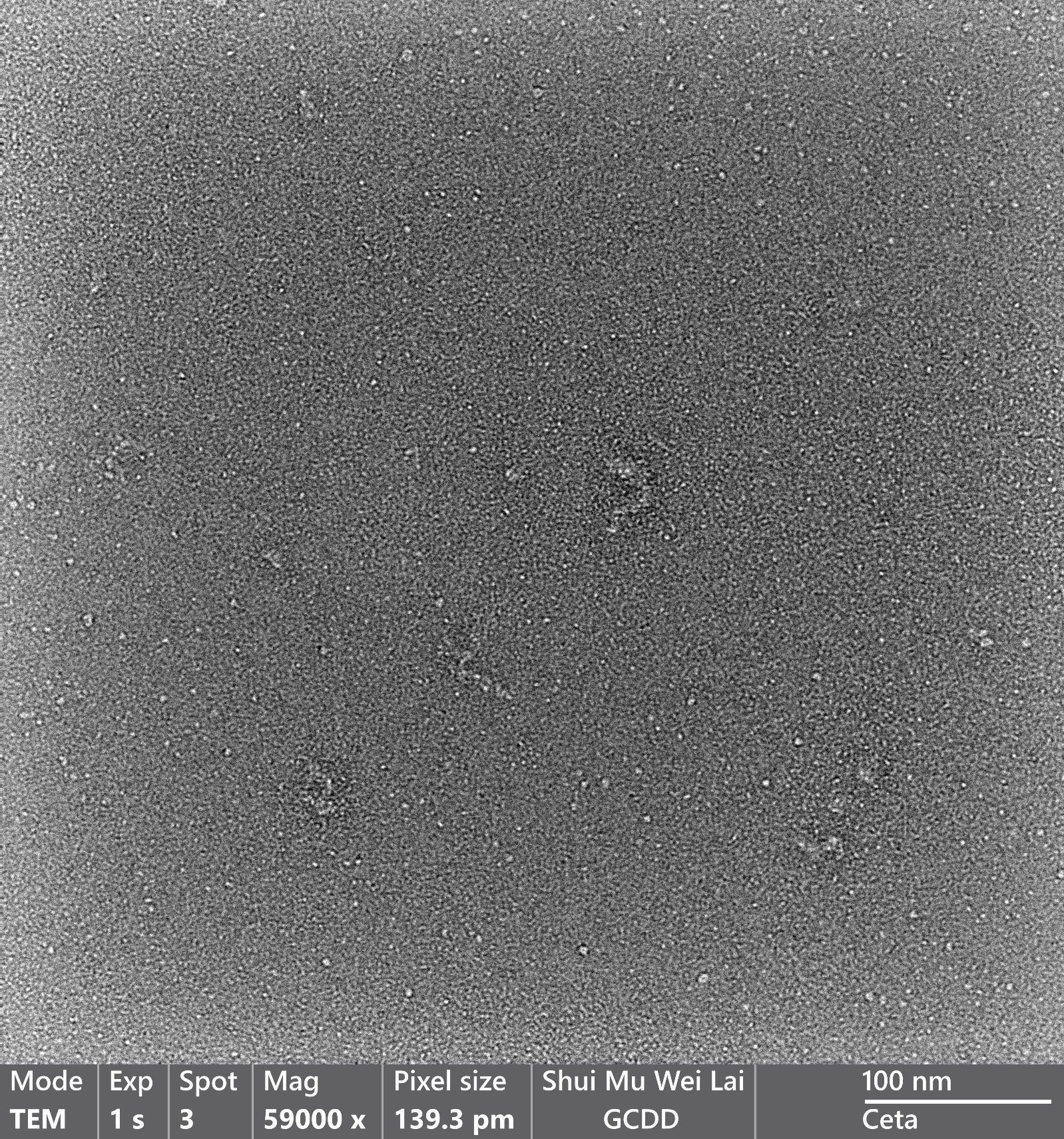


**Figure S7 AML12-Bright:**


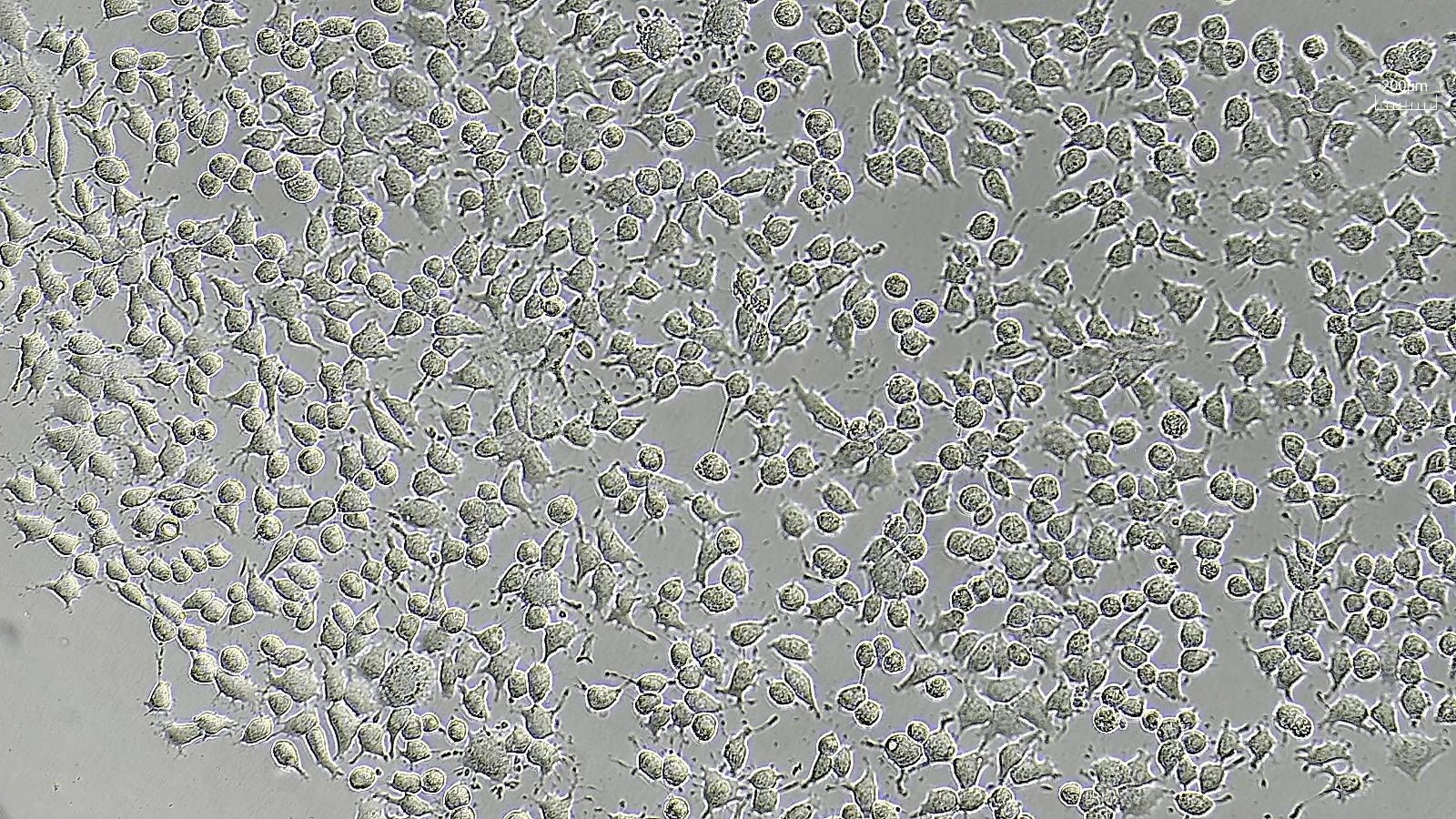


**Figure S7 RAW264.7-Bright:**


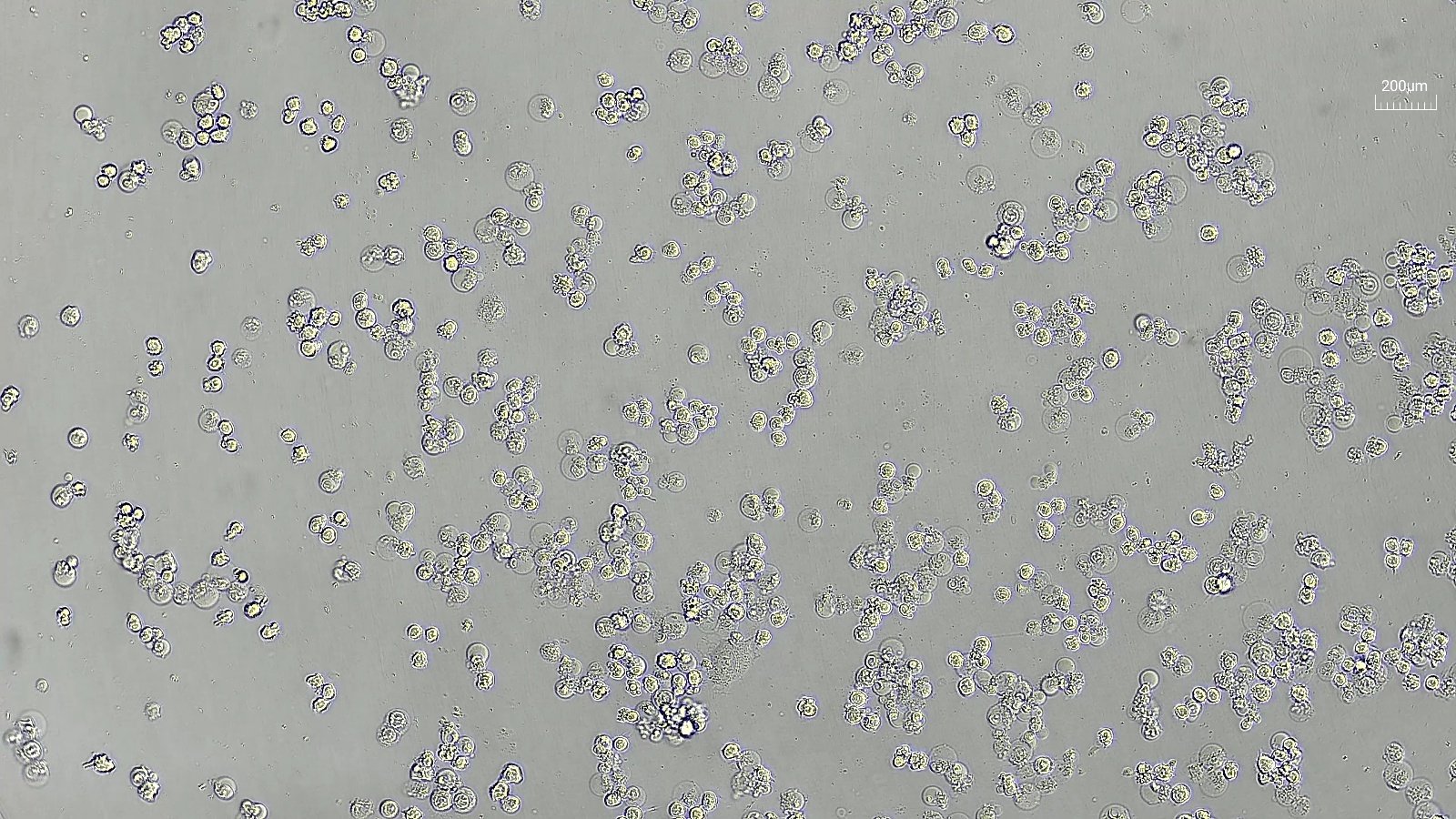


**Figure S7 GES-1-Bright:**


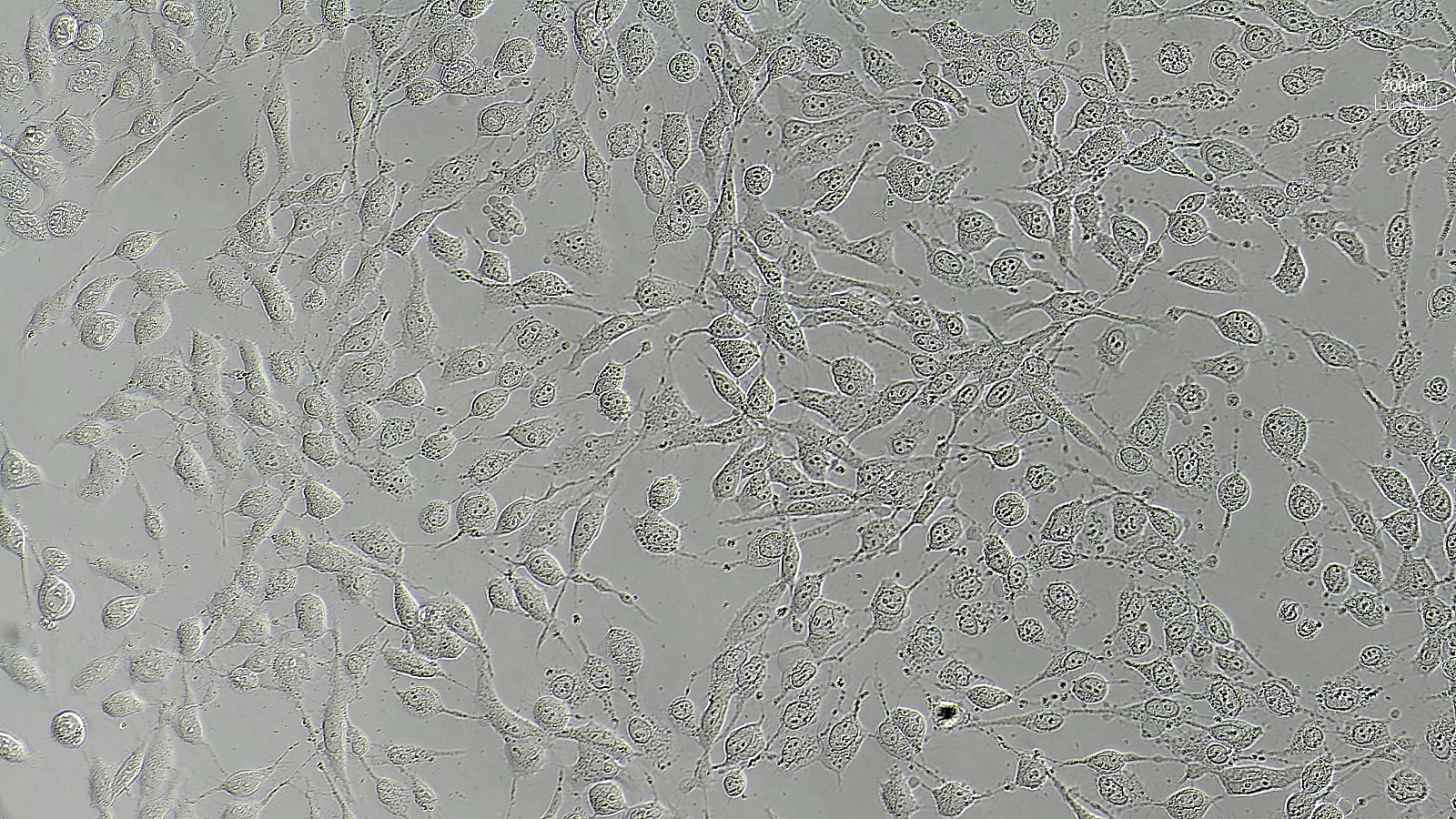


**Figure S7 AML12-Hoechst:**


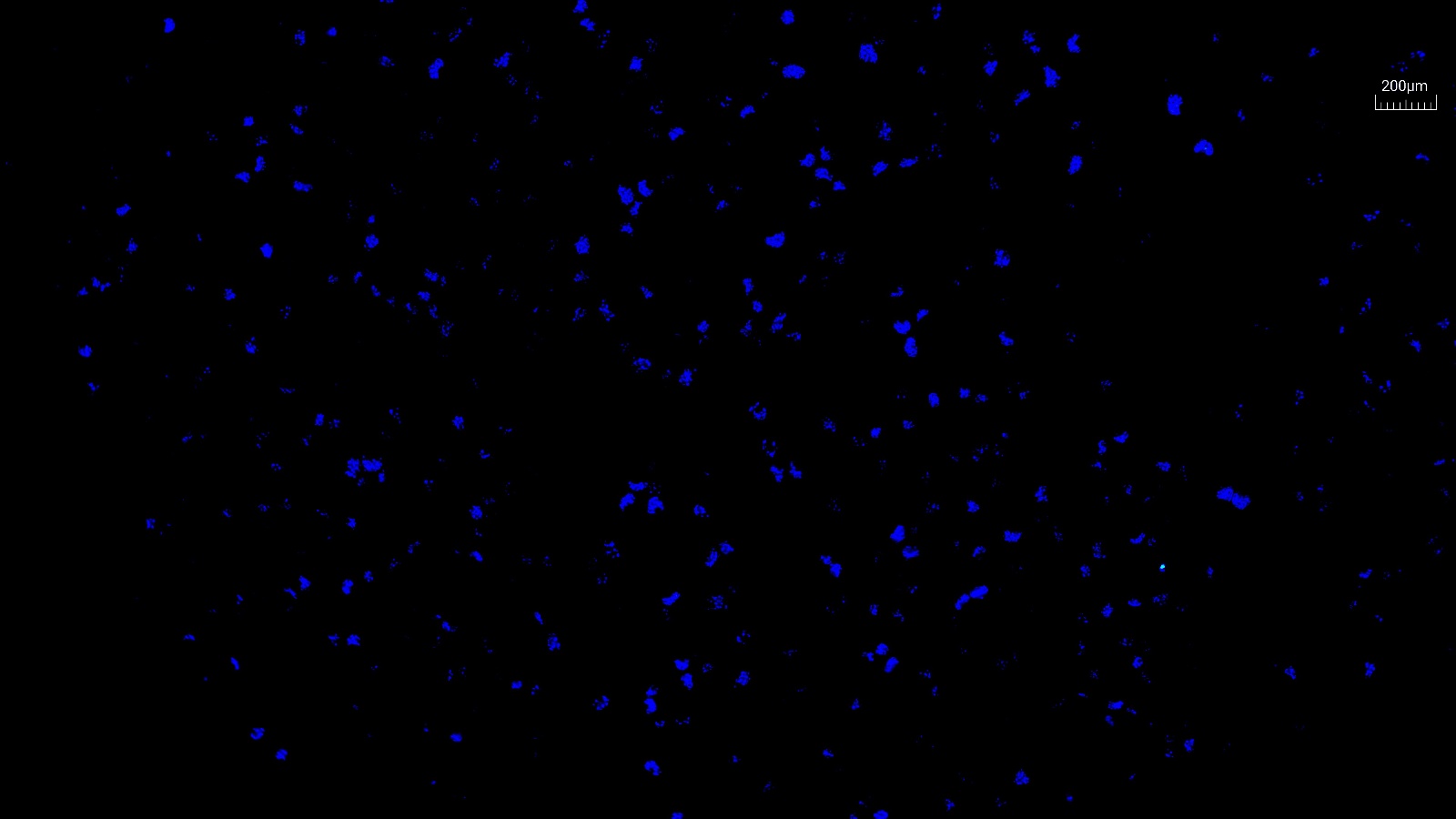


**Figure S7 RAW264.7-Hoechst:**


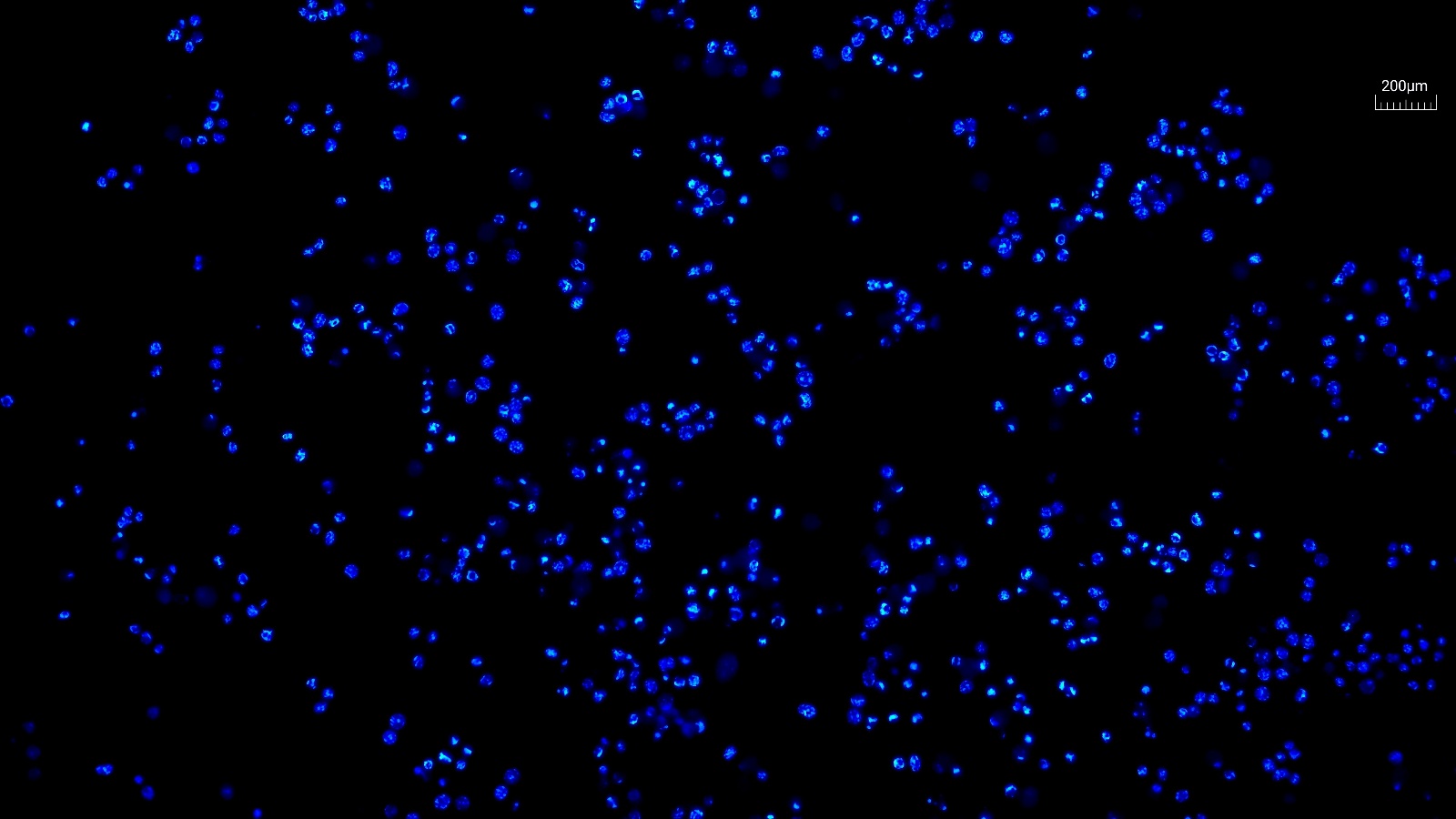


**Figure S7 GES-1-Hoechst:**


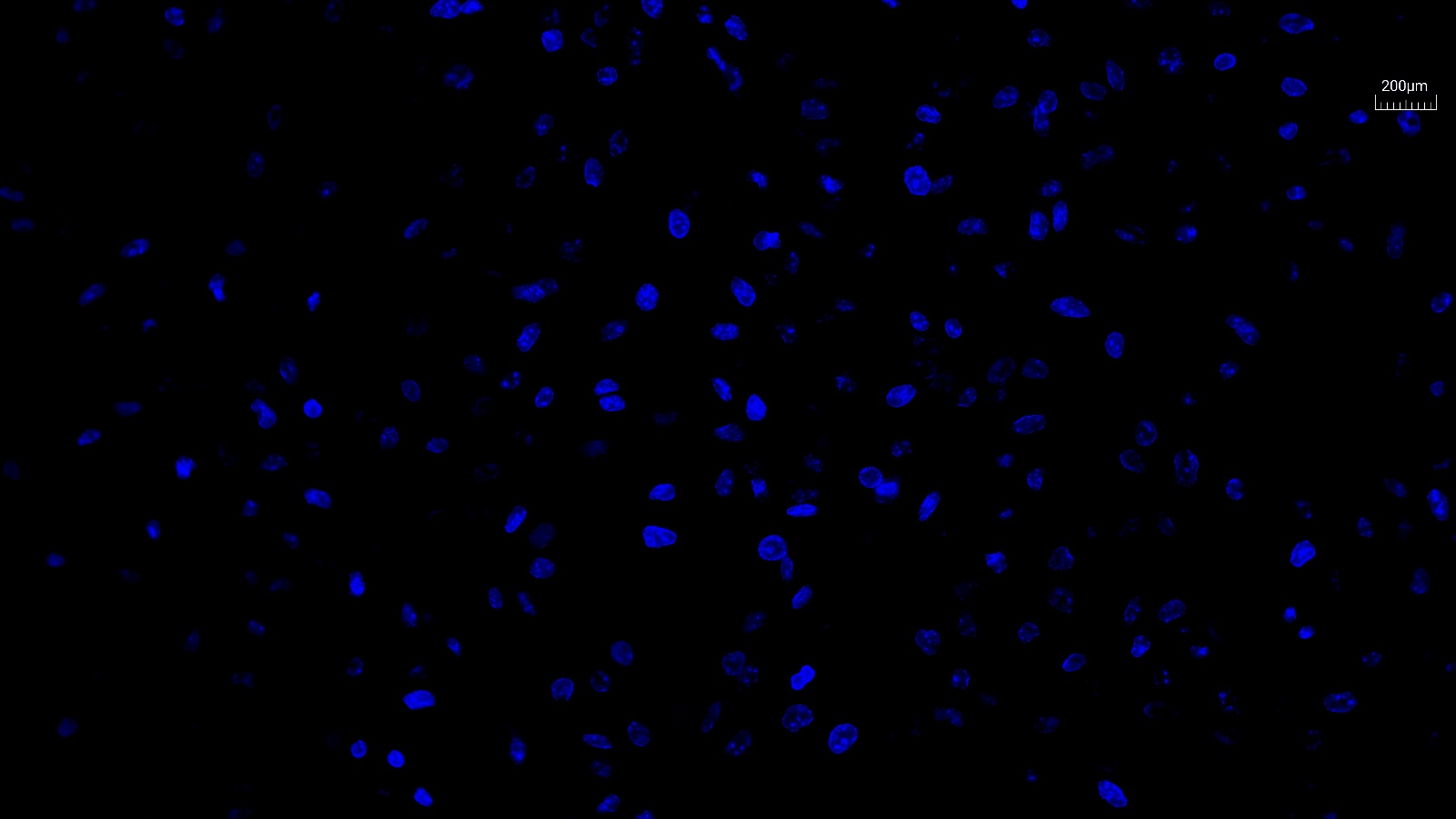


**Figure S7 AML12-Lyso tracker (Green):**


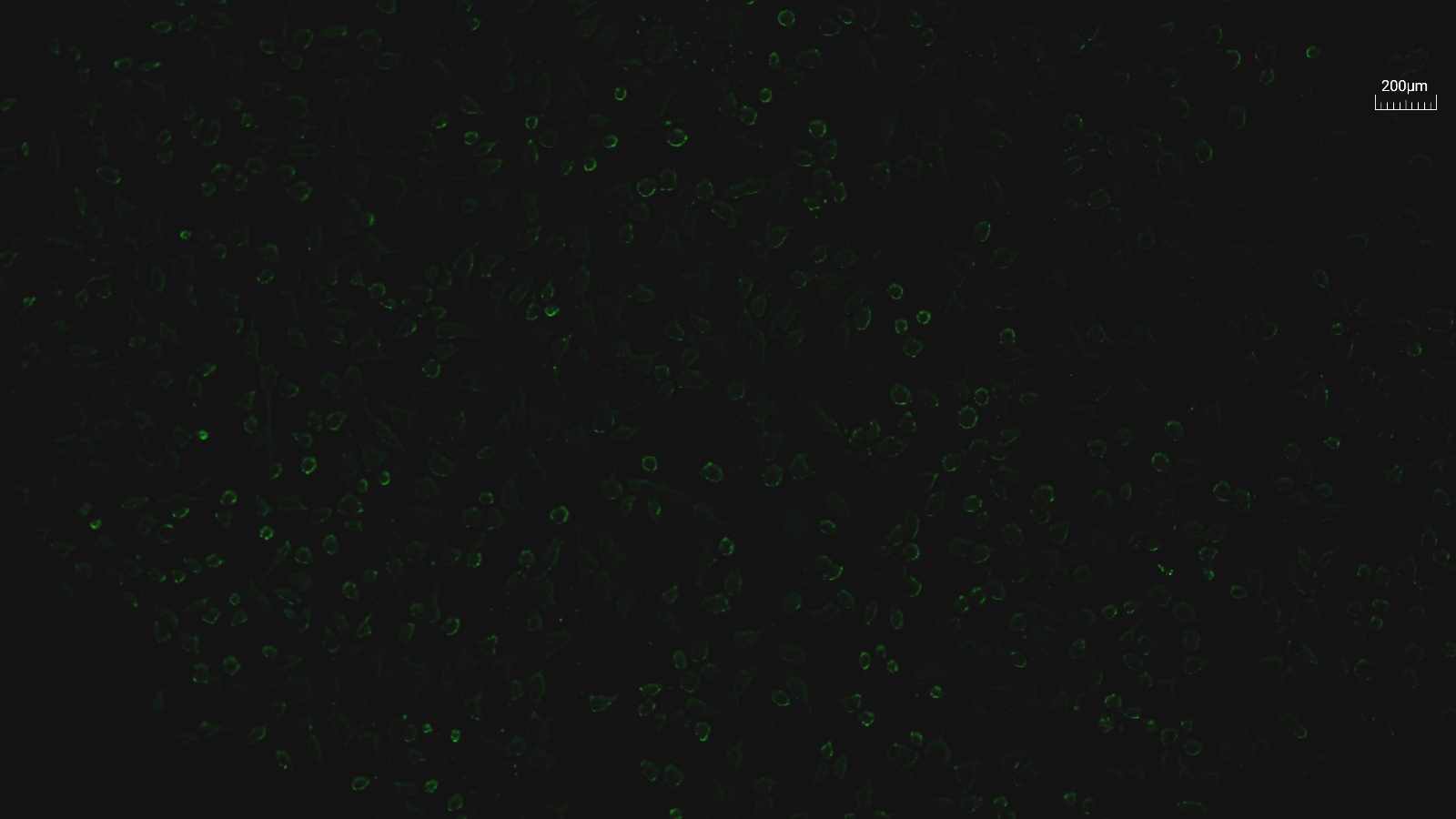


**Figure S7 RAW264.7-Lyso tracker (Green):**


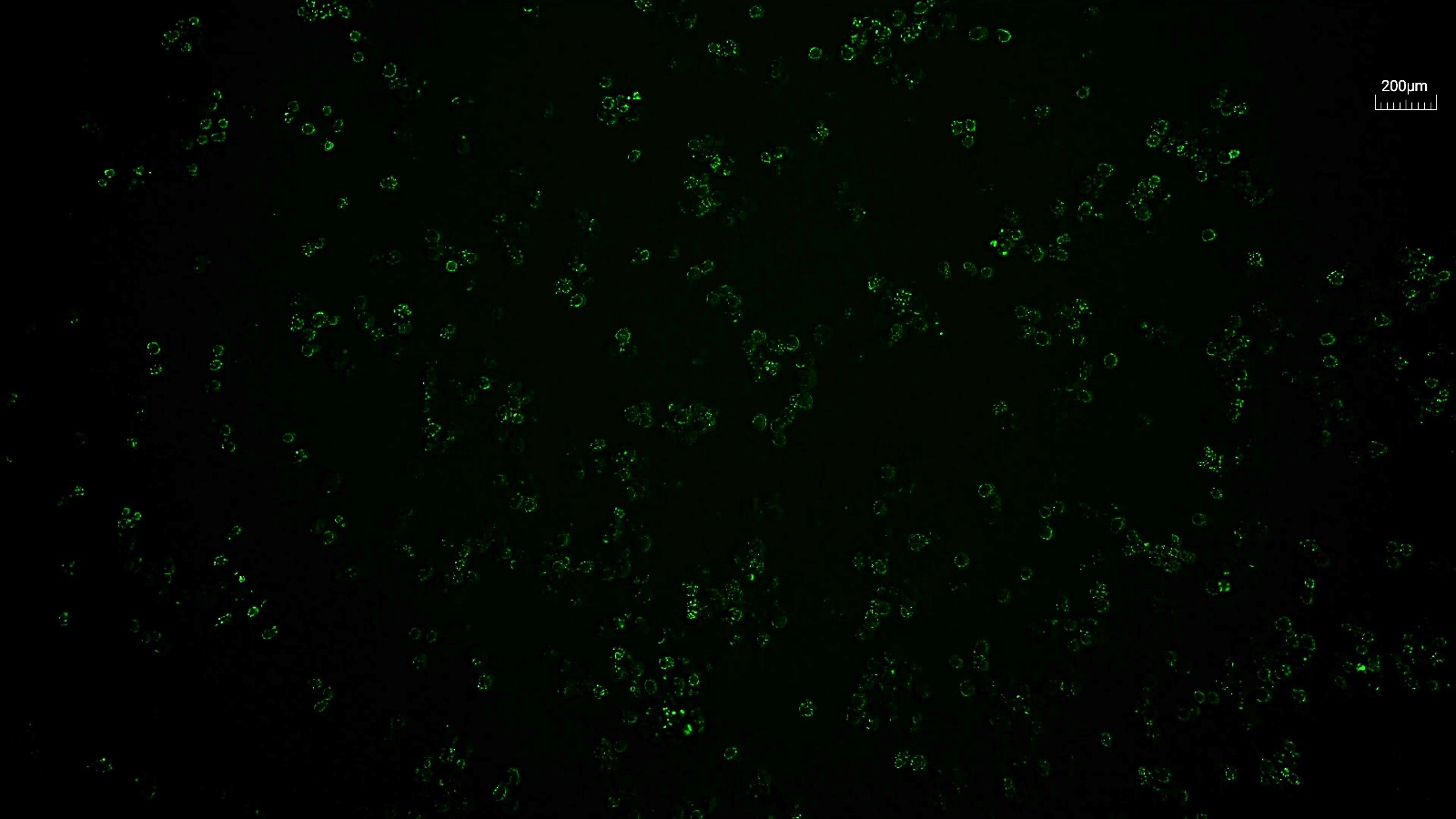


**Figure S7 GES-1-Lyso tracker (Green):**


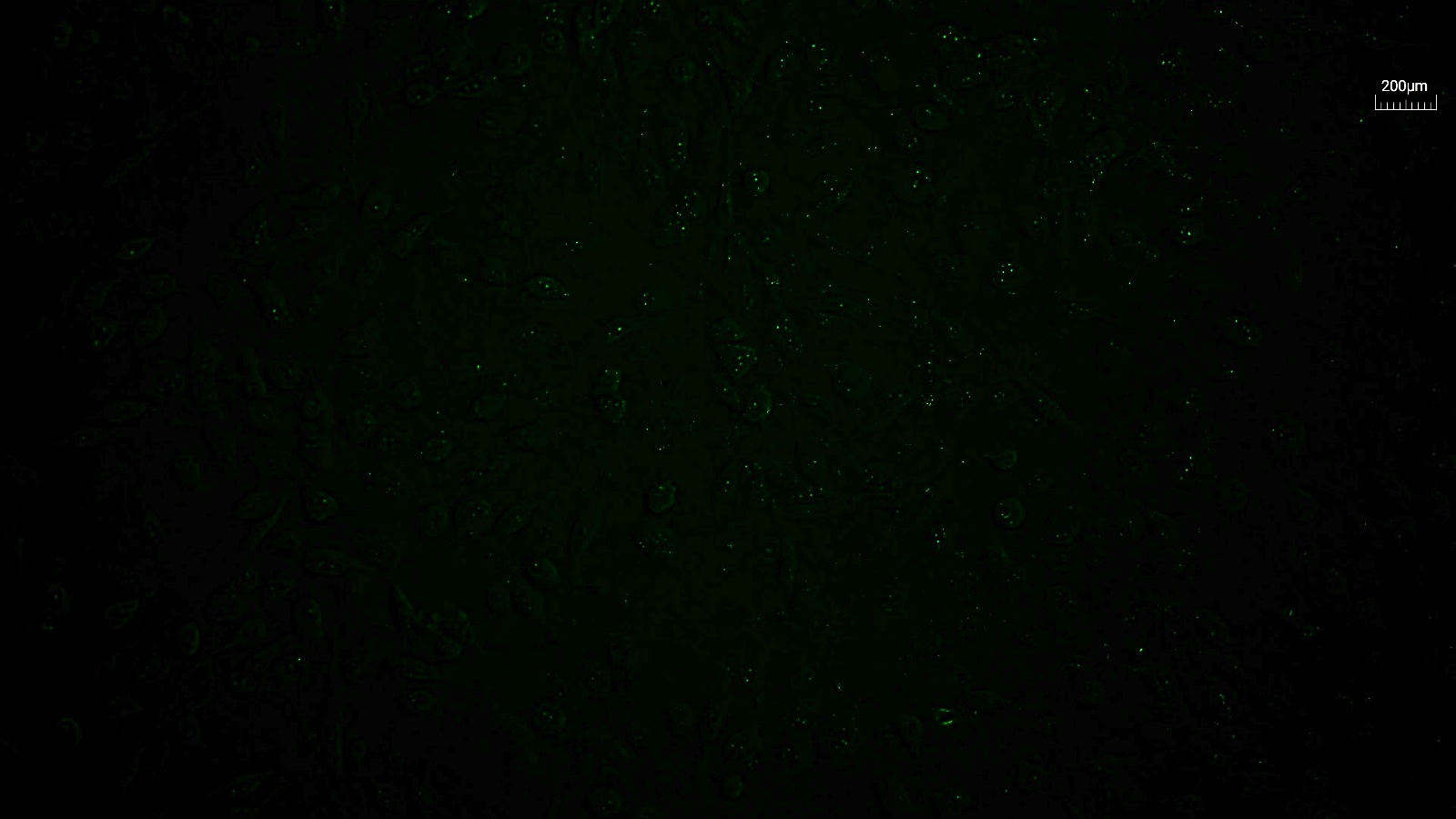


**Figure S7 AML12-CY5-labeled targeted tFNAs:**


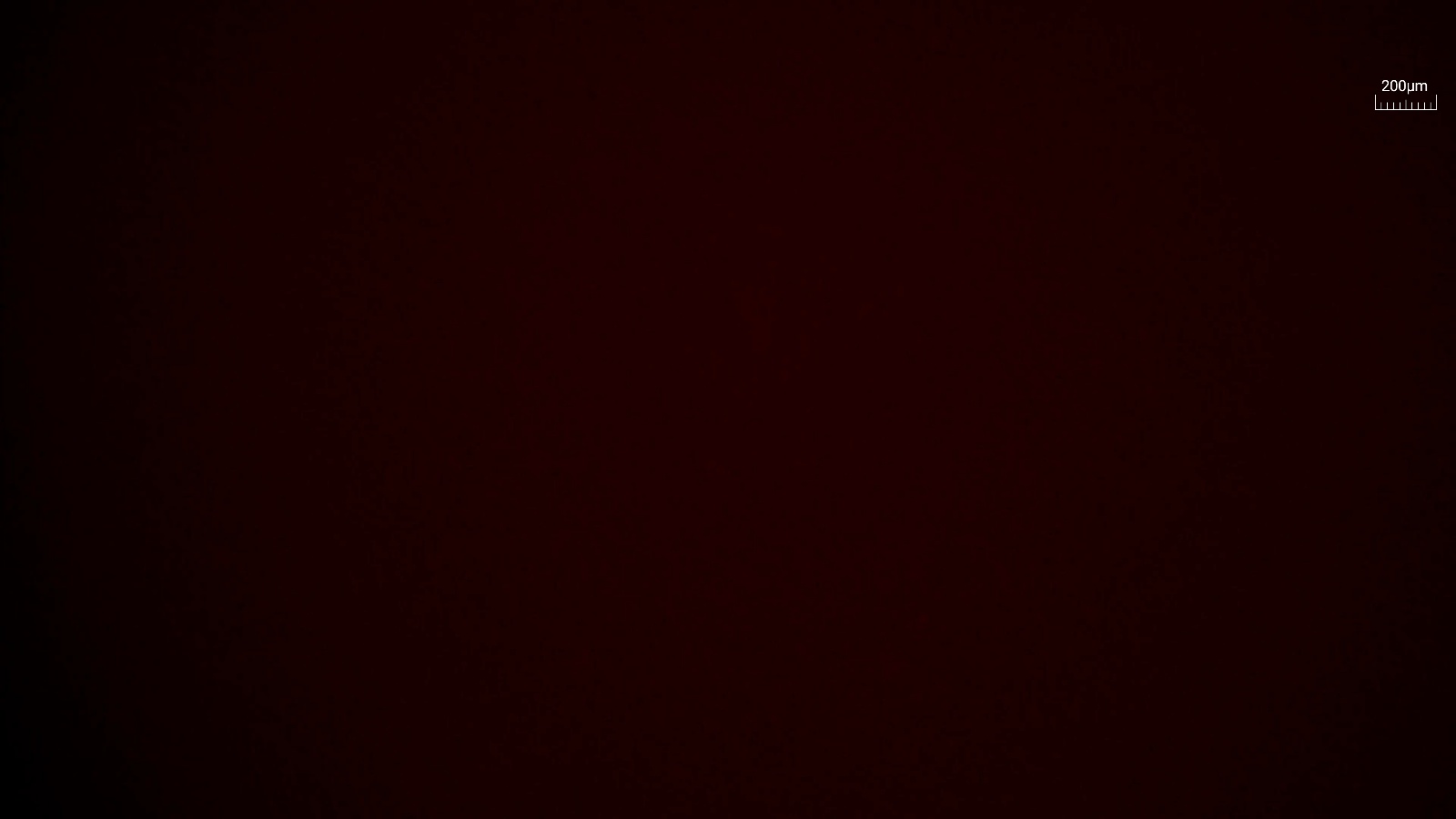


**Figure S7 RAW264.7-CY5-labeled targeted tFNAs:**


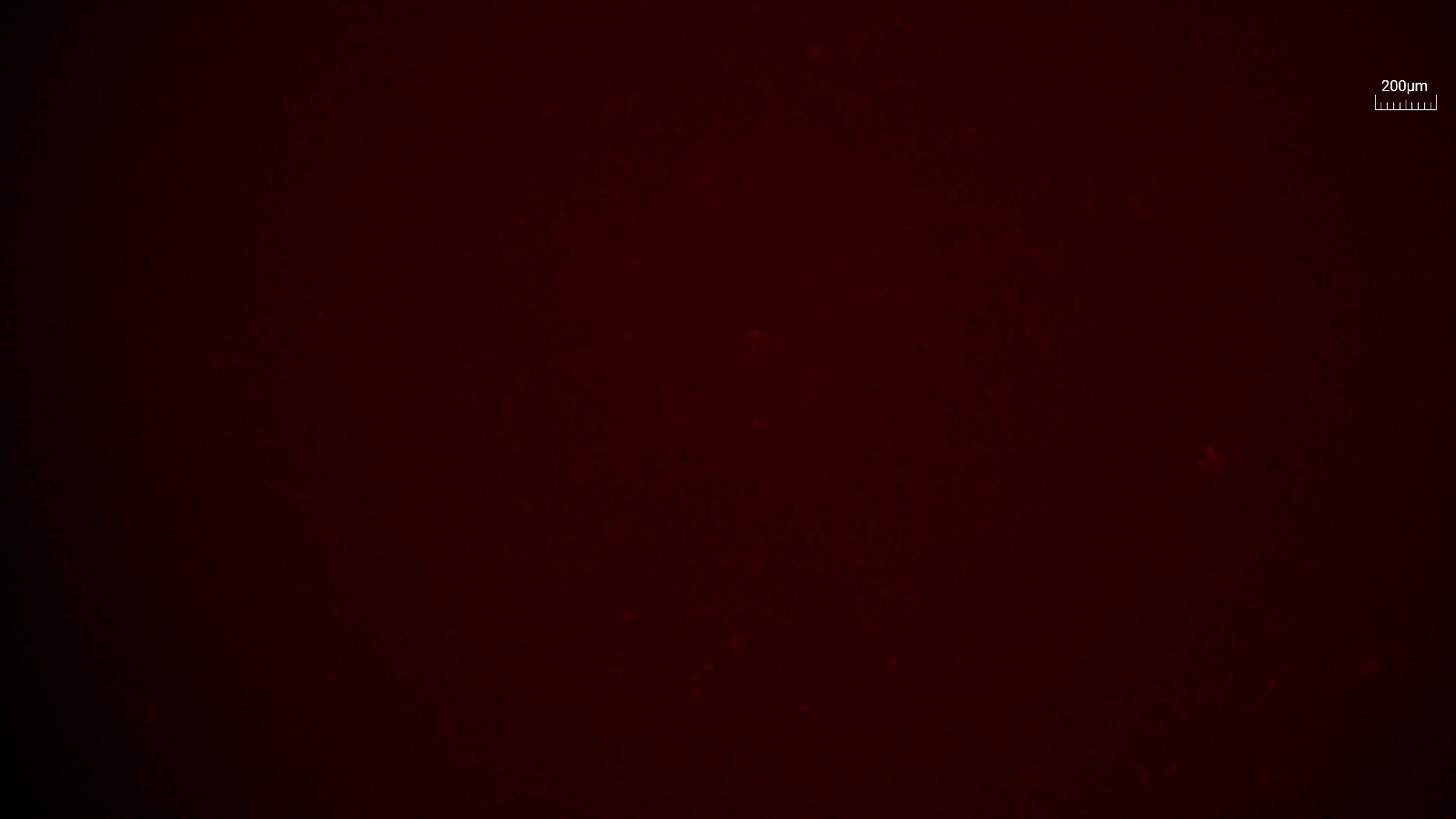


**Figure S7 GES-1-CY5-labeled targeted tFNAs:**


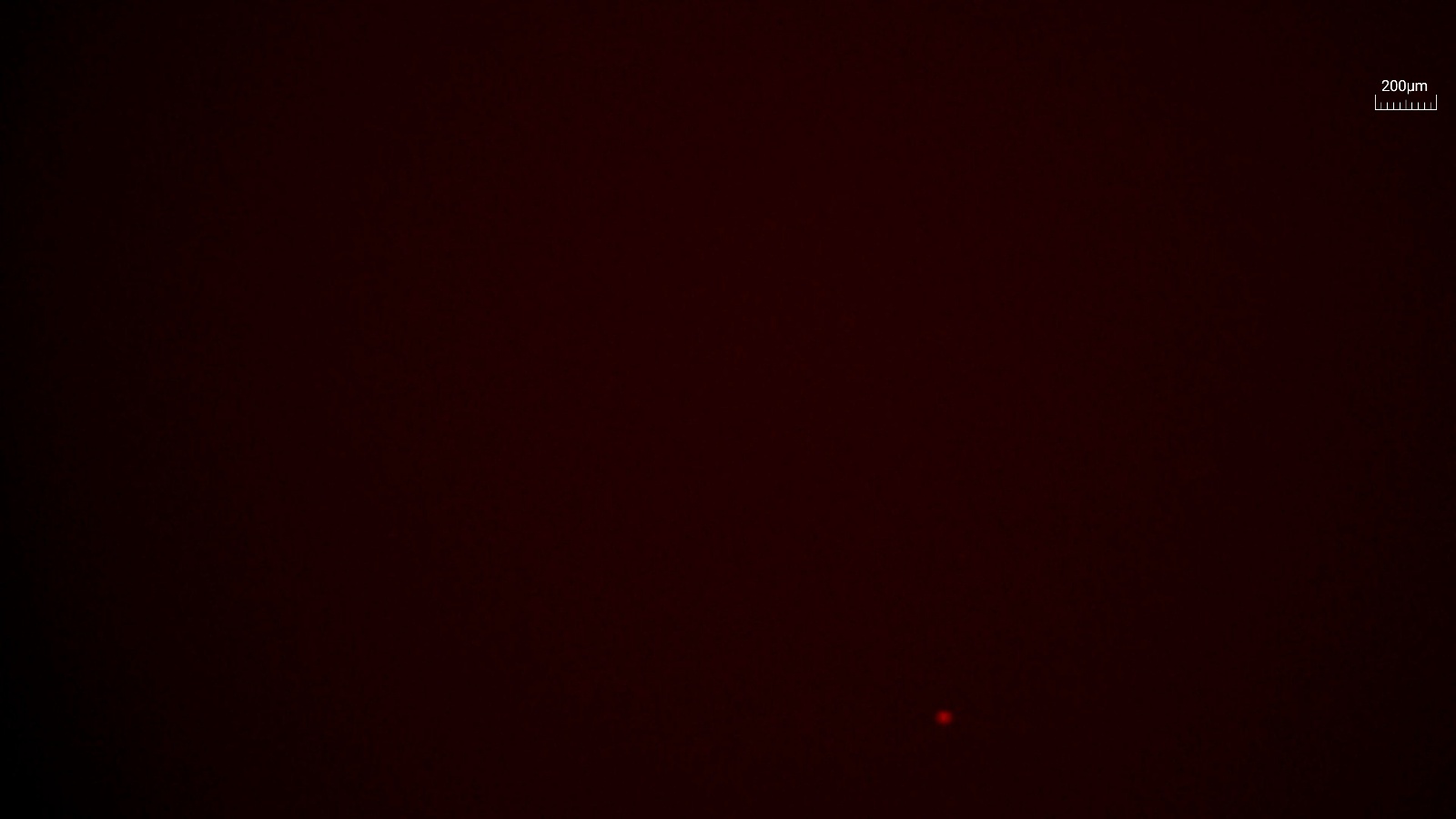


**Figure S7 AML12-Merge:**


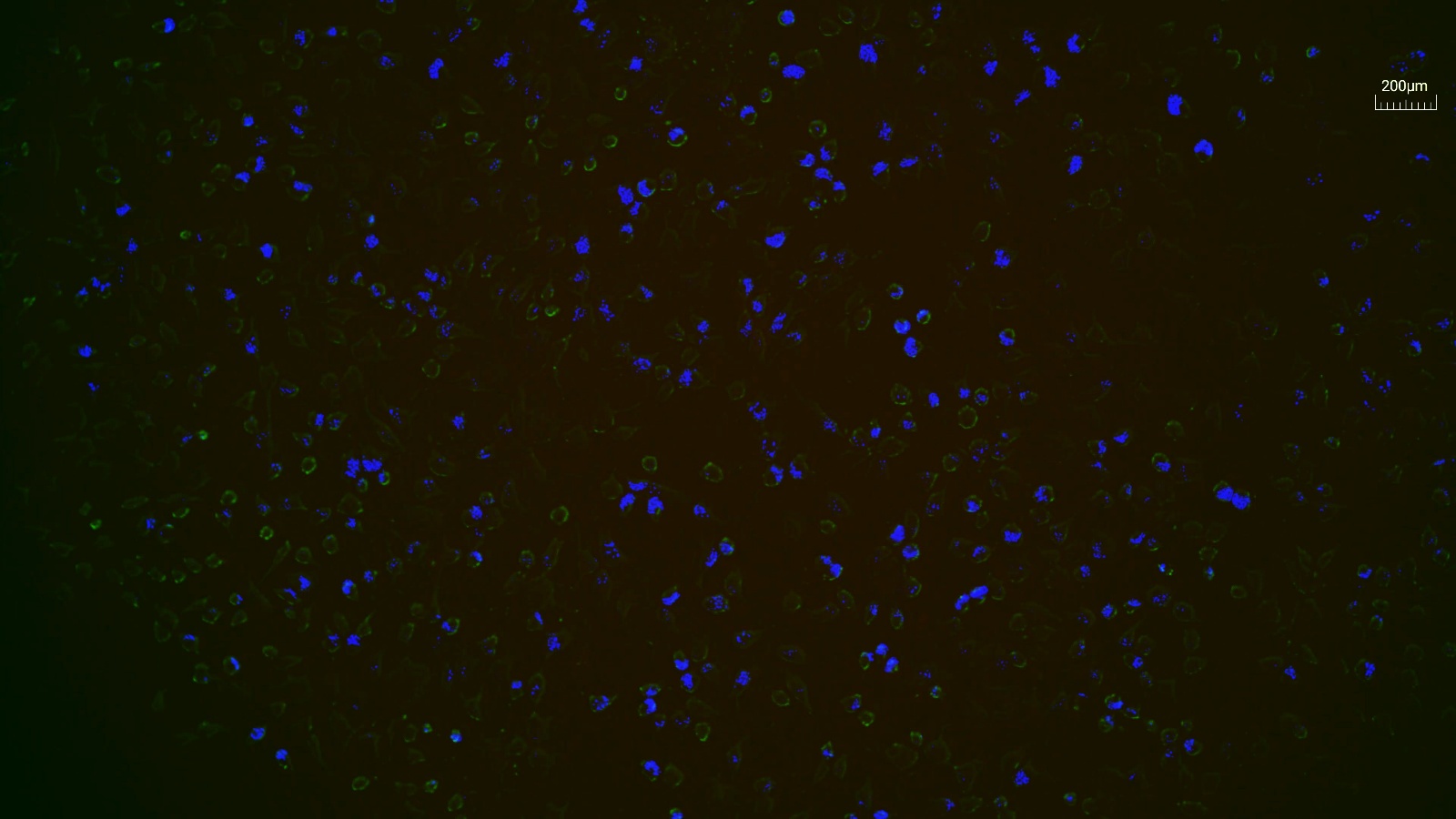


**Figure S7 RAW264.7-Merge:**


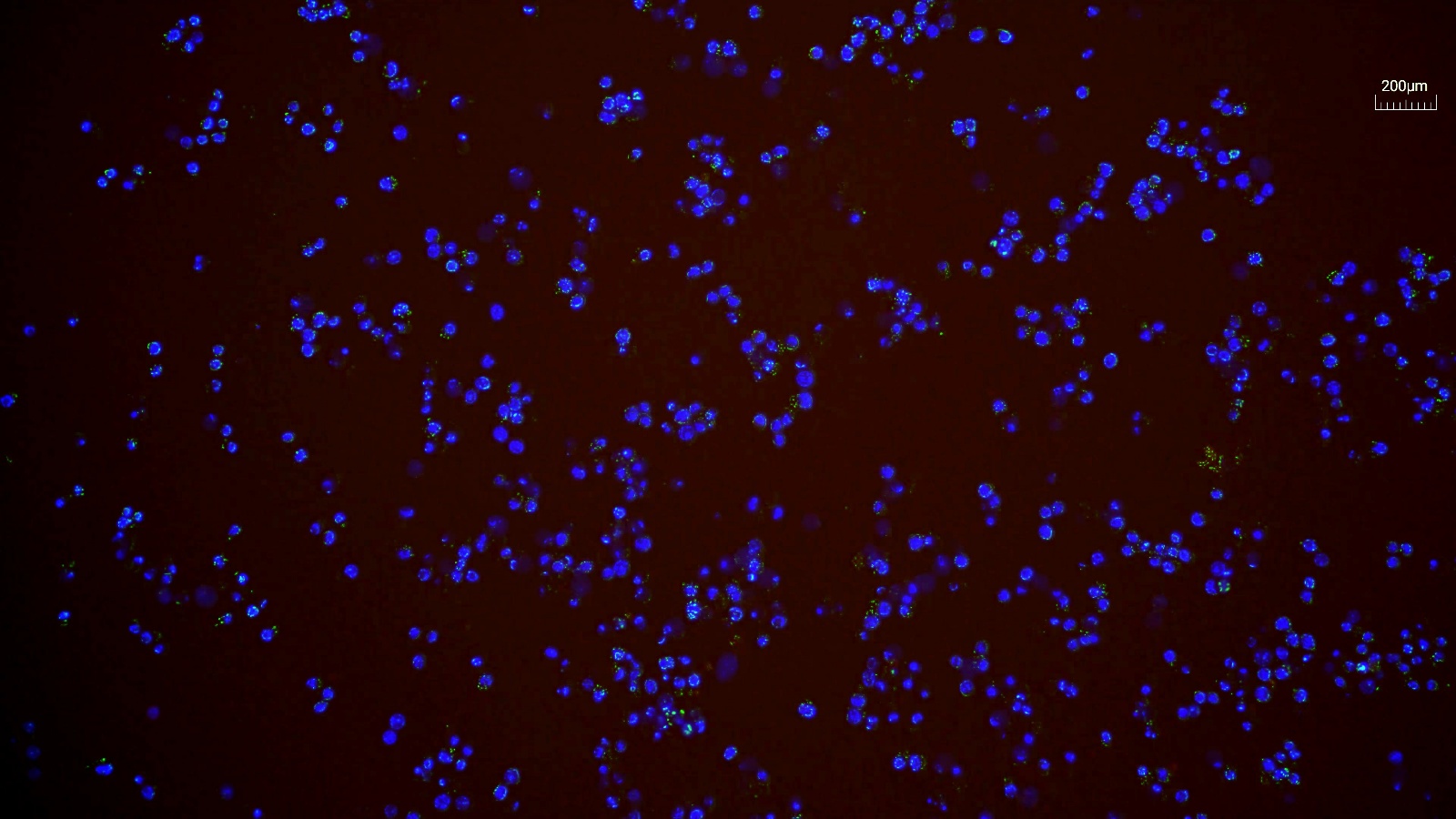


**Figure S7 GES-1-Merge:**


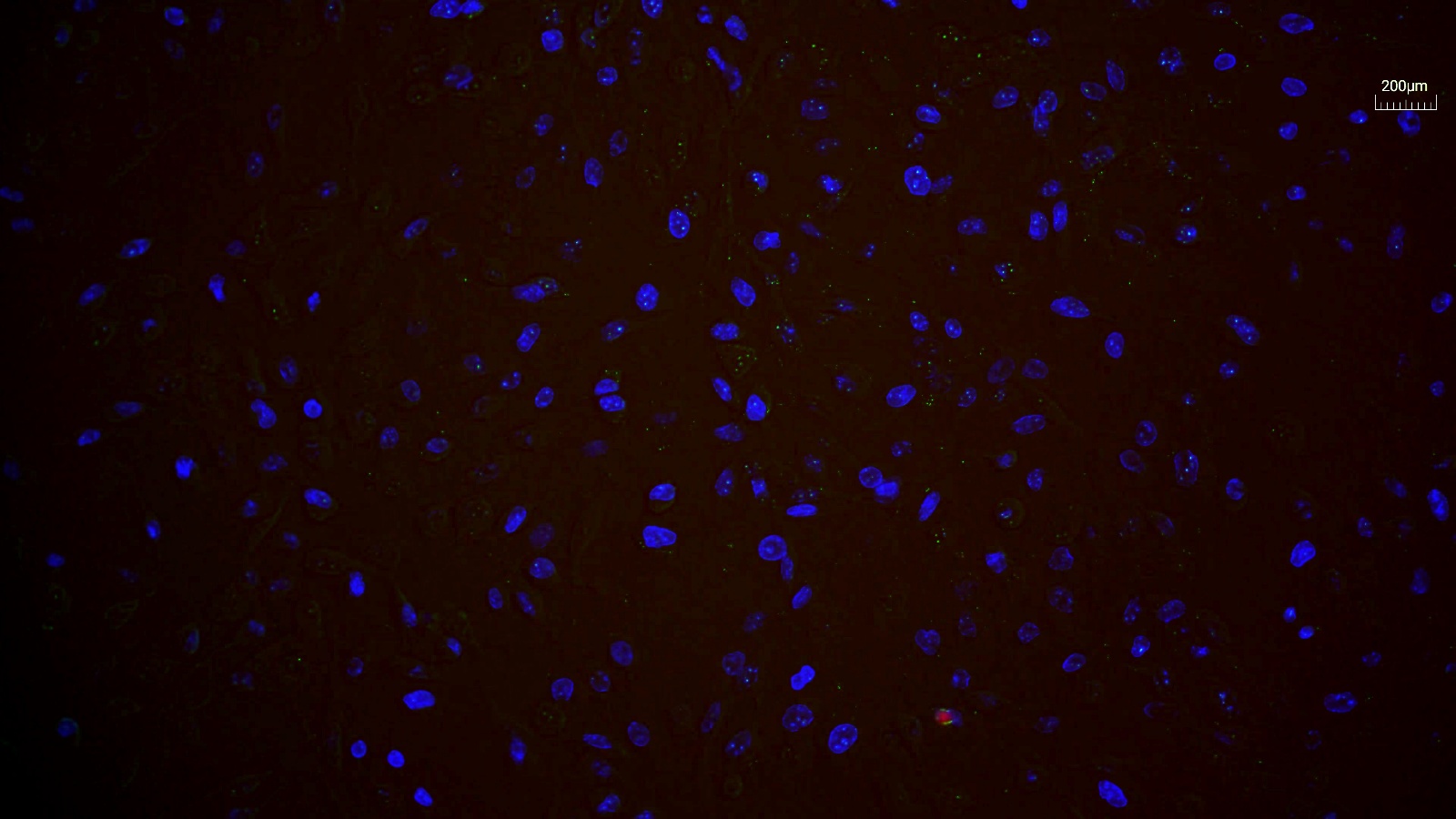

Supplement: Supplementary Material — Figure S4 TEM photographs of untargeted tFNAs: [file IDRD_A_2576222_SM3787.docx]
